# Exposure route mediates toxicological effects of sulphur and fluxapyroxad fungicides in a non-target butterfly

**DOI:** 10.1371/journal.pone.0353528

**Published:** 2026-07-09

**Authors:** Mine Yilmazer, Marie Bakenecker, Roland Busch, Farina Fuchs, Anna Hitzler, Klaus Fischer

**Affiliations:** Department of Biology, Institute for Integrated Natural Sciences, University of Koblenz, Universitätsstraße 1, Koblenz, Germany; Charles University: Univerzita Karlova, CZECHIA

## Abstract

Agricultural intensification and the associated increase in pesticide use have raised concerns about impacts on non-target insects. However, fungicides, despite their frequent application, remain poorly studied, particularly for herbivorous species. This is partly caused by the fact that they are considered less harmful than other pesticides. We here investigate the effects of three fungicides commonly used in viticulture, two sulphur- and one fluxapyroxad-based ones, on the cabbage white butterfly (*Pieris rapae*) under controlled laboratory conditions. Larvae were exposed to fungicides orally via treated host plants or by direct contact, and mortality and sublethal effects were assessed during development. In our study, oral exposure did not increase mortality relative to controls. Regarding sublethal effects, larvae exposed orally to sulphur-based fungicides exhibited a longer pupal development compared to controls. Additionally, larvae exposed to fluxapyroxad showed shorter development time and higher fat content than those exposed to sulphur-based fungicides. Contact exposure resulted in increased mortality relative to controls, with no significant differences among fungicides. These findings demonstrate that fungicide effects on non-target Lepidoptera are route-dependent, resulting in sublethal impacts via ingestion and lethal effects via contact exposure. Our results thus emphasize the need to consider (1) the dual risk posed by fungicides through both lethal and sublethal effects, (2) exposure route, and (3) fungicide-specific responses when assessing fungicide risks, particularly in agricultural systems where non-target Lepidoptera may occur. Detrimental effects of direct contact can be minimized through the timing of fungicide applications, thereby reducing the route-dependent risks identified in this study.

## 1. Introduction

Modern agriculture relies heavily on plant protection to prevent yield losses caused by pests and plant pathogens. Over the past decades, the global use of pesticides has increased dramatically, reaching unprecedented levels across various cropping systems [1,2]. Today, around 3.7 million tonnes of pesticides are applied worldwide each year [3]. While these substances clearly play an important role in securing agricultural production, their increased use has raised concerns about potential impacts on human health and non-target organisms [4,5]. A growing body of evidence suggests widespread declines in insect abundance and diversity across different ecosystems [6,7]. Because insects contribute substantially to ecosystem functioning, these declines have become a major concern for both biodiversity conservation and sustainable agriculture [8,9]. Among the multiple drivers discussed, increasing pesticide use is likely to be a key contributing factor [10–12].

Viticulture is one of the most intensively managed agricultural systems worldwide, relying heavily on the application of plant protection [13,14]. Several fungal pathogens are known to infect grapevines, requiring repeated fungicide applications throughout the growing season [13]. As a result, fungicides typically account for most pesticide treatments in European vineyards, frequently involving 12–15 applications per year [13,14]. Consequently, potential impacts of fungicides on non-target organisms may be particularly relevant for species inhabiting vineyards. Vine-growing regions may comprise biodiversity hotspots [15–17], exaggerating potential conflicts. One prominent example is the Mosel Apollo (*Parnassius apollo vinningensis*), a highly specialised and critically endangered butterfly, which occurs in or close to vineyards where its larval host plant, *Sedum album*, is present [18–20]. Its dramatic decline in recent years has been documented in long-term monitoring data and coincide with the increasing use of modern fungicides in its habitat, raising concerns about a potential contribution of fungicide exposure to population declines, although direct evidence is currently lacking [19]. Non-target organisms may be exposed to fungicides via direct contact or oral uptake. Direct contact may occur during or immediately after spraying, while oral uptake occurs through consumption of contaminated host plants [21].

Despite the widespread use of fungicides in agriculture, their effects on non-target insects have received little attention and remained, consequently, poorly understood [1,21,22]. This is at least partly because they are generally considered less harmful to arthropods than, e.g., insecticides [22]. However, growing evidence indicates that fungicides may have substantial negative effects on non-target organisms [23,24]. Most ecotoxicological research on fungicides to date has focused on predatory arthropods such as spiders or mites, or on beneficial insects like honeybees (*Apis mellifera*), which can exhibit measurable declines in abundance or fitness under fungicide exposure (e.g., [14,25–28]). In contrast, herbivorous insects, making up roughly half of all insect species, have received even less attention [29]. As important regulatory risk assessments, including current EU insecticide registration protocols, do not consider this ecologically important group, we are facing a critical gap in understanding the broader ecological impacts of pesticide use [21,24]. This underscores the urgent need to incorporate herbivorous insects into regulatory risk assessments. Some recent studies on butterflies, an important group of herbivorous insects, indicate that fungicide exposure can cause substantial negative effects on development, metabolism, and survival [24,30,31].

Fungicides differ widely in their chemical properties and modes of action, which can result in varying impacts on non-target organisms [22,23]. Sulphur is one of the oldest fungicides and remains widely applied to control powdery mildew [13,32]. Sulphur acts mainly as a contact fungicide and is generally considered to have low ecotoxicity for non-target species, including bees, earthworms, and other soil macro-organisms [33]. However, some studies have reported adverse effects of sulphur on certain invertebrates (e.g., [34,35]). In addition to such traditional contact fungicides, systemic fungicides have been developed to improve disease control, e.g., succinate dehydrogenase inhibitor (SDHI) fungicides, which are now widely used in conventional agriculture [36,37]. These fungicides inhibit mitochondrial respiration in fungi by blocking the enzyme succinate dehydrogenase. Yet, as similar mitochondrial pathways are conserved across eukaryotes, they may potentially affect all organisms with mitochondria [38,39]. Such systemic fungicides, including fluxapyroxad, may cause toxic and sublethal effects in various non-target organisms, inducing developmental abnormalities, oxidative stress, and behavioural alterations (e.g., [39–42]). Taken together, these findings suggest that ecological risks of fungicides are currently likely underestimated, especially with regard to herbivorous insects.

We here investigate the effects of three fungicides often used in viticulture, two sulphur-based contact fungicides and the systemic SDHI fungicide fluxapyroxad. We applied contact and oral exposure to evaluate acute and sublethal effects on the cabbage white butterfly, *Pieris rapae* (Pieridae). Including sublethal effects seems important, as current risk assessments mainly focus on acute toxicity endpoints [33] and may thus overlook more subtle effects. In particular, we investigate whether (i) fungicide exposure affects survival and development relative to untreated controls, (ii) the tested fungicides differ in acute toxicity following oral and / or contact exposure, (iii) potential effects depend on the uptake pathway (oral vs. contact). We predict that fungicide applications will have detrimental effects on survival and development, with fluxapyroxad being more detrimental than sulphur-based fungicides.

## 2. Materials and methods

### 2.1 Study organism

We used the cabbage white *P. rapae* (Pieridae) to investigate the effects of fungicide exposure on a non-target organism. This species is highly sensitive to pesticides [43,44], making it an appropriate model organism for ecotoxicological studies. *P. rapae* occurs widely in agricultural and semi-natural landscapes. Its larvae feed on a broad range of Brassicaceae, including crops such as cabbage, rapeseed, and turnips, which exposes them frequently to agrochemicals including fungicides [45–47]. The species is multivoltine, producing typically 3–4 generations per year in Central Europe; it overwinters as pupa [48]. The combination of high sensitivity to agrochemicals, wide distribution, and ecological relevance makes *P. rapae* a suitable model species for studying lethal and sublethal effects of fungicides.

*P. rapae* is not a protected species under German nature conservation law; therefore, no collection permits were required for the present study. All applicable institutional and national guidelines for the care and use of animals were followed.

### 2.2 Study area, population sampling and oviposition

In July and August 2025, we collected 25 fecund females of *P. rapae* in the vicinity of Koblenz (50.356° N, 7.588° E; supplementary information, S1 Table: sampled females, catch date, location, collection method). This area has a suboceanic climate with mild average temperatures of around 10.8 °C and 674 mm mean precipitation annually [49]. Caught females were transported to the University of Koblenz and maintained in a climate chamber (Memmert HPP260) set to 26 °C, 60% relative humidity, and a photoperiod of L18:D6 (light from 5 a.m. to 11 p.m. [30,50]). Each female was kept individually in a gauze‑covered plastic container (1 L) with a fresh leave of young cabbage (Brassica oleracea var. capitata) for oviposition. Females were fed a 10% sucrose solution and nectar plants. The latter included *Lavandula angustifolia*, *Leucanthemum vulgare*, *Daucus carota* and *Achillea millefolium*. Daily, eggs were collected and host plant leaves, nectar plants, and sucrose solution replaced. Resulting eggs were transferred to glass vials with perforated lids, containing young cabbage leaves for larval feeding. After hatching, larvae were transferred to 250 mL plastic boxes, with a maximum of 10 larvae per box, lined with a moist paper towel and containing cabbage leaves. 24 hours prior to the start of the experiments, larvae were individually transferred to 250 mL plastic rearing boxes to assess larval condition (feeding activity, digestion, mobility), enabling selection of suitable individuals for the experiment. Thus, larvae that were inactive, possibly moulting to the final larval instar or in poor condition, were not included in the experiments. Throughout, all individuals were maintained under the conditions outlined above for oviposition. For the experiments, larvae of each treatment were housed in separate, identical climate chambers (Memmert HPP260) to prevent cross-contamination, and the lids of the rearing boxes were replaced with gauze until pupation to allow air circulation and prevent accumulation of volatile compounds such as sulphur. Cabbage was purchased from a local organic market in Koblenz, Germany (Demeter-certified production). However, exposure to plant protection products permitted under organic farming regulations cannot be fully excluded, which would affect all treatment groups though.

### 2.3 Experimental design

#### 2.3.1 Oral exposure experiment.

On day 8 after hatching, larvae were distributed across treatments using a split-brood design to avoid potential lineage effects. To investigate the effects of fungicides, we used four treatment groups: (1) control (no fungicide), (2) Stulln® (sulphur-based fungicide; 800 g/kg elemental sulphur), (3) Thiovit Jet® (sulphur-based fungicide; 800 g/kg elemental sulphur), and (4) Sercadis® (succinate dehydrogenase inhibitor fungicide; active ingredient: fluxapyroxad; 300g/L formulation), with ca. 100 individuals per treatment. Cabbage leaves were sprayed with fungicides, completely dried under a fume hood (approximately 45 minutes) before being fed to the larvae or stored for longest 5 days at 4°C before being fed. Leaves were provided ad libitum in small sections (approximately 7 × 10 cm, variable shape). After ten days in their respective treatment, all larvae were subsequently fed untreated cabbage leaves until pupation. Paper and leaves were replaced daily.

Fungicide treatments were designed to mimic helicopter applications as used in conventional viticulture [51]. The tested products are ones typically applied during the larval activity of *P. apollo vinningensis* (May / June; [19,20]) in the Mosel region. Therefore, cabbage leaves were arranged on a 1 m² area within a 1 m³ cage, to ensure a consistent application height of 1 m (supplementary information, S1A Fig). We applied 100 mL solution per m² using a Gloria 3 hand-held sprayer, which produces a fine spray fog. A separate sprayer was used for each treatment to avoid cross-contamination, and sprayer were calibrated before experiments (see supplementary information: S1 Text, S2 Fig, S2 Table). Doses were scaled to one square-metre, resulting in 0.4  g/m² for the sulphur formulations and 0.012  mL/m² for Sercadis® (supplementary information, S3 Table). To determine the doses applied to leaves per 1 m², six petri dishes (90 mm diameter) were placed on the spray area for each treatment (supplementary material, S1B Fig). The dishes were weighed before and immediately after spraying, and the difference in liquid volume was extrapolated to 1 m² using the formula: dose per m² = (applied amount in dish / dish area) × 1 m², from which active ingredient concentrations per cm² of leaf area (µg/cm²) were subsequently calculated (see supplementary information, S3 Table). No residue analysis of leaf surfaces was performed; thus, exposure estimates are based on gravimetrically determined depositions rather than direct measurements of leaf surface concentrations.

#### 2.3.2 Contact exposure experiment.

On day 9 or 10 after hatching, larvae were sprayed with different fungicides (Stulln®, Thiovit Jet®, Sercadis®) and concentrations, using 10 larvae each. For each fungicide, we used eight concentrations. First, a stock solution of 100%, corresponding to 10 times the maximum authorized field rate, was prepared for each compound, resulting in 80 g/L for the sulphur-based fungicides and 16 mL/L for Sercadis®. This high concentration was used to ensure a sufficient dose-response range to capture potential toxic effects. The stock solution was then diluted in six steps (supplementary information, S4 Table), and additionally a control treatment (0%) with water only was included. The maximum field rates for sulphur were derived from the manufacturers’ product specifications (Stulln®, Nufarm; Thiovit Jet®, Syngenta), whereas the rate for fluxapyroxad (Sercadis®) was taken from the Federal Office of Consumer Protection and Food Safety list of plant protection products authorized for helicopter application [52]. For spraying, each larva was placed on a piece of paper towel within a pre-marked area (10 x 13 cm) of a plastic box (supplementary information, S3 Fig). Solutions were applied from approximately 40 cm above the larva using a single pump stroke each. Throughout the contact test, larvae were fed untreated cabbage leaves; paper towels and leaves were replaced daily. To determine the applied doses (supplementary information, Table S4), one petri dish (90 mm diameter) per fungicide and concentration was placed in the predefined exposure area and sprayed with one pump stroke (supplementary information, S2 Text, S4 Fig). Sprayers were calibrated prior to experiments (supplementary information, S2 Text).

### 2.4 Data analysis

For all individuals in both experiments we scored survival, and, if applicable, larval and pupal development times in days as well as larval growth rate as mass gain per day (LN pupal mass / larval developmental time). Resulting pupae were weighed to the nearest 0.01 mg (Sartorius CPA225D) one day after pupation. Eclosed adults were frozen at −18 °C for further analyses. To this end, wings, head, and legs were removed. Thorax and abdomen were separated and weighed (Sartorius CPA225D) to calculate the thorax-abdomen ratio, representing the relative investment into flight versus reproduction. Furthermore, forewing length was measured using digital images of the left forewings, using ImageJ (FIJI). To assess abdominal fat content, we followed the procedure described in Fischer et al. ([53]). Briefly, abdomens were dried at 60 °C for 24 h to obtain initial dry mass. Fat was then extracted twice for 48 h each using 1.6 mL dichloromethane (CH₂Cl₂) per extraction. Then, abdomens were dried and weighed again to obtain the fat-free dry mass. Fat content was calculated as the difference between initial and fat-free dry mass and is given as relative fat content (fat content / abdomen mass × 100).

### 2.5 Statistical analyses

All statistical analyses were conducted in R (version 4.3.1; [54]) using the packages Lme4 [55], Emmeans [56], DHARMa [57], Car [58], Survival [59], and Survminer [60].

#### 2.5.1 Oral exposure experiment.

Mortality (0/1) was analysed using generalized linear mixed-effects models (GLMMs) with a binomial error distribution and a logit link. Treatment was included as fixed factor, and maternal identity as random effect to account for the non-independence among siblings. Model fit was evaluated using DHARMa residual simulations to assess uniformity, overdispersion, and outliers. Significance of fixed effects was tested using a Type III ANOVA (Wald χ² tests). Differences among treatments in survival probability were analysed using Cox proportional hazards, and survival curves for each treatment were visualised using the Kaplan-Meier method. Sublethal effects were analysed using linear mixed-effects models (LMMs) with Gaussian error distribution. Treatment and sex were included as fixed factors, and maternal identity as random effect. Prior to modelling, Shapiro-Wilk tests were applied to assess normality, and traits with significant deviations were LN-transformed (larval and pupal developmental time, thorax mass, thorax-abdomen ratio, wing length). LMMs were assessed using Type III ANOVA with Satterthwaite approximation to evaluate the effects of treatment, sex, and their interaction. Significant treatment effects were further explored using pairwise comparisons of estimated marginal means (EMMeans). Model fit for LMMs was checked using residual diagnostic plots (DHARMa).

#### 2.5.2 Contact exposure experiment.

Mortality (0/1) was analysed using generalized linear mixed effects models (GLMMs) with a binomial error distribution and a logit link. Treatment and concentration were included as fixed effects, and maternal identity as random effect. LC50 estimations could not be performed due to the absence of a consistent monotonic concentration-response relationship in the data. Model fit was evaluated using DHARMa residual simulations. Pairwise post-hoc comparisons of fixed effects were conducted using estimated marginal means (EMMeans). Survival probability was analysed using Cox proportional hazards to assess the effects of treatment, concentration, and their interaction. Survival curves for each treatment were visualised using the Kaplan-Meier method. In both analyses, data were analysed including and excluding controls (0%) to rule out that potential differences among fungicides are masked by controls. As patterns were very similar in both respective analyses, only the results including controls are reported. In this data set, sublethal effects were not analysed due to high mortality rates and concomitantly highly unbalanced sample sizes.

## 3. Results

### 3.1 Oral exposure experiment

**Fig 1:**
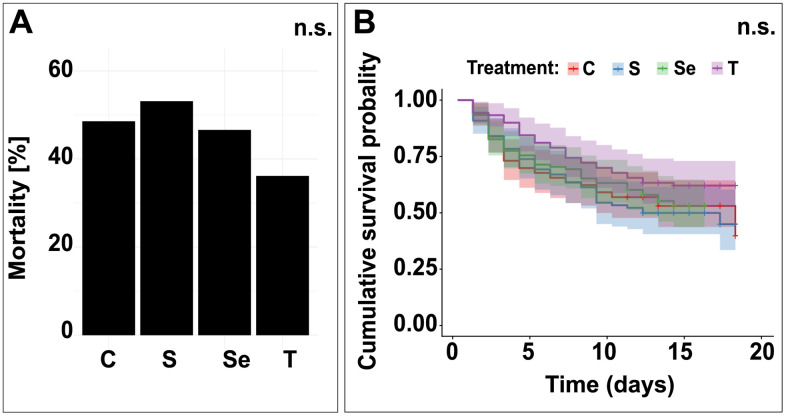
Mortality rates (A) and cumulative survival probability (B) of *Pieris rapae* after oral exposure to different treatments (C: control, S: Stulln®, Se: Sercadis®, T: Thiovit Jet®). In (B), survival probability is presented from the allocation to treatments (day 8) onwards and ranges from 0 to 1, with values closer to 1 indicating a higher likelihood of survival. (A) and (B) are scored until adult eclosure.

**Fig 2:**
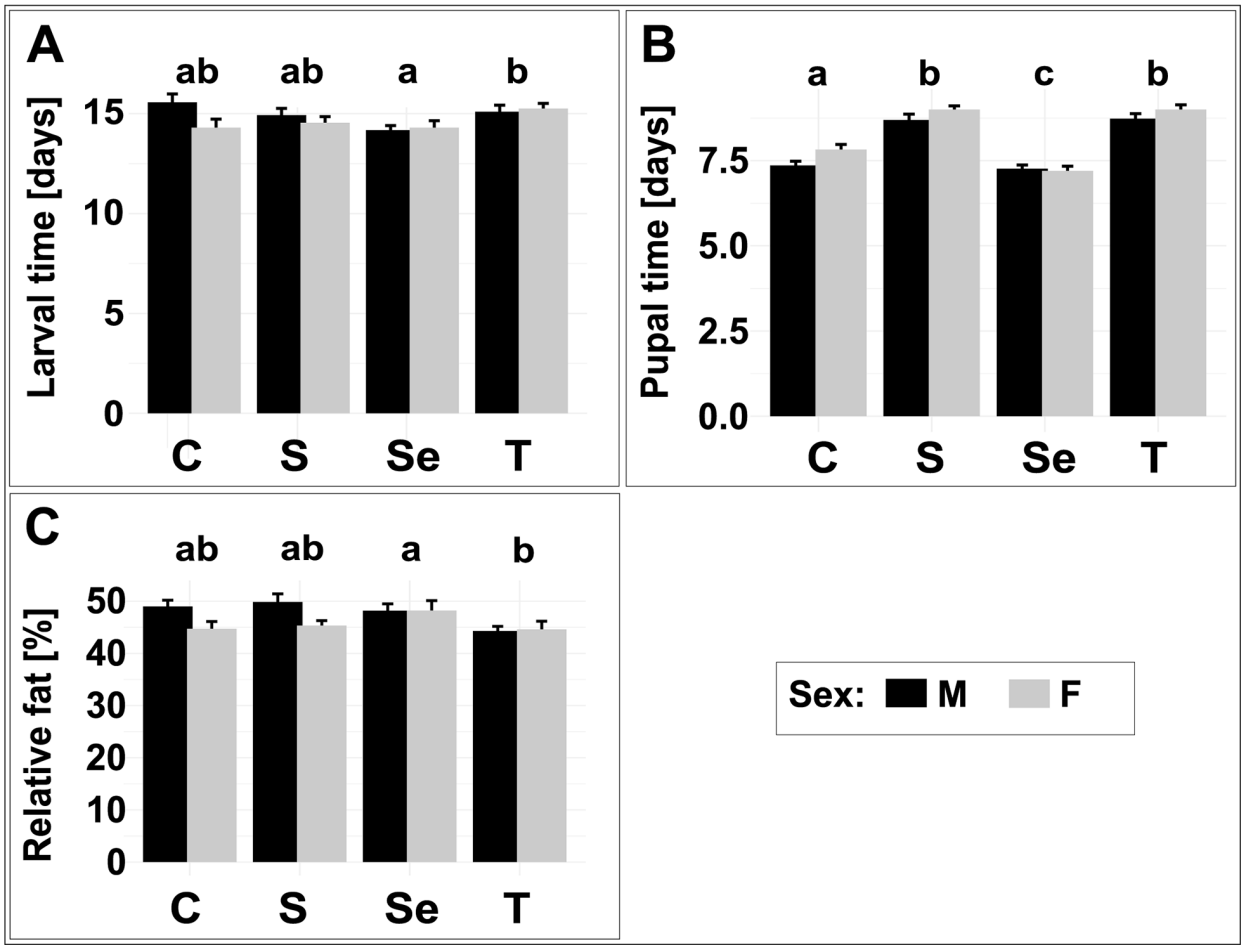
Effects of treatment (C: control, S: Stulln®, Se: Sercadis®, T: Thiovit Jet®) and sex on (A) larval developmental time, (B) pupal developmental time, and (C) relative fat content in *Pieris rapae* after oral exposure. Given are means ± SE. Different letters above bars indicate significant differences among treatments (supplementary information, S1 and S2 Tables).

Mortality ranged between 36 and 53% (Fig. 1A; Table 1) with no significant differences across treatments (χ²_3_ = 5.03, p = 0.170). Likewise, treatment had no significant effect on survival probability (Cox proportional hazards; χ²₃ = 4.96, p = 0.175; Fig 1B). Significant sublethal treatment effects were observed for development times (larval: F_3,194_ = 3.46, p = 0.018, pupal: F_3,195_ = 83.39, p < 0.001) and relative fat content (F_3,177_ = 3.78, p = 0.012; supplementary information, S5 and S6 Tables). Individuals treated with Sercadis® showed shorter larval times than those treated with Thiovit Jet®, and also shorter pupal times compared to all other treatment groups. In contrast, pupal times in individuals treated with Stulln® or Thiovit Jet® were particularly long (Fig 2). Relative fat content was higher in the Sercadis® than in the Thiovit Jet® treatment. Significant sex differences were detected for pupal development time (F_1,198_ = 4.86, p = 0.029), pupal mass (F_1,193_ = 8.78, p = 0.003), and relative fat content (F_1,179_ = 3.98, p = 0.047; supplementary information, S5 and S7 Tables). Males had shorter pupal times (males: 8.0 ± 0.2 d; females: 8.2 ± 0.2 d), higher pupal masses (males: 126.6 ± 3.4 mg; females: 120.8 ± 3.4 mg), and higher relative fat contents than females (males: 48.0 ± 1.6%; females: 46.1 ± 1.6%). The sex by treatment interactions were not significant throughout.

**Table 1:** Survival of *Pieris rapae* under oral exposure treatments.

Treatment	n	Survivors (n)	Survival %	Longevity ± SD
Control	99	51	51.5	22.55 ± 2.02
Stulln®	98	46	46.9	23.61 ± 1.78
Sercadis®	101	54	53.5	21.46 ± 1.41
Thiovit Jet®	97	61	63.5	24.32 ± 2.21

Overview of treatments for oral exposure in *Pieris rapae*, showing initial sample size, surviving individuals, survival probability, and mean longevity until death.

### 3.2 Contact exposure experiment

**Fig 3:**
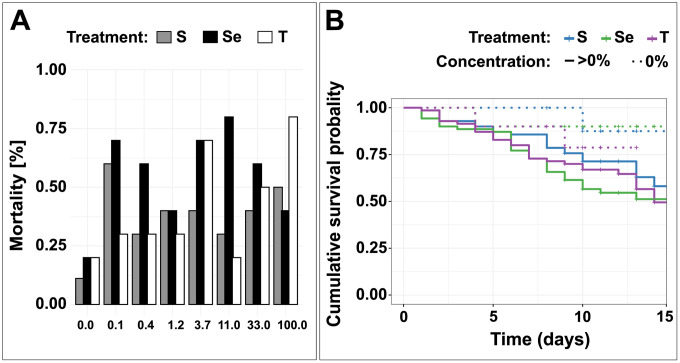
Mortality rates (A) and cumulative survival probability (B) of *Pieris rapae* larvae after contact exposure to different fungicides (S: Stulln®, Se: Sercadis®, T: Thiovit Jet®) and concentrations (0%−100%). In (A), mortality is shown per treatment and concentration based on n = 10 individuals per combination, resulting in a single value per group. In (B), the 0% concentration (i.e., no active ingredients) serves as reference compared to all other concentrations. Survival probability is presented from the allocation to treatments onwards (day 9 / 10) and ranges from 0 to 1, with values closer to 1 indicating higher likelihood of survival.

Generalized linear mixed models revealed a significant increase in mortality with increasing concentration (χ²_1_ = 4.32, p = 0.038), while no significant differences among treatments were found (χ²_2_ = 5.89, p = 0.053; interaction n.s.; Fig 3A). These patterns were confirmed by a survival analysis showing significant effects of concentration (Cox Proportional Hazards; χ²_7_ = 21.49, p = 0.003), with survival being highest in controls (0%; Fig 3B; supplementary information, S8 Table), while no significant treatment effect was found (χ²_2_ = 1.96, p = 0.376; interaction n.s.).

## 4. Discussion

Although fungicides are widely used in agriculture, only few studies have examined their effects on non-target insects. In particular, the risks for herbivorous insects are understudied, despite evidence that fungicides may detrimentally affect development, metabolism, energy reserves, or survival [1,21,24,30,31]. Also, studies so far mainly focussed on oral exposure [24,30,31], while even less is known about contact exposure, which may be ecologically particularly relevant as evidenced by our results.

### 4.1 Oral exposure: no acute toxicity but subtle sublethal effects

Under the given experimental conditions, ingestion of fungicide residues via treated host plants did not cause acute toxicity, i.e., survival was unaffected. Likewise, monarch (*Danaus plexippus*) larvae fed leaves treated with different fungicides (azoxystrobin, pyraclostrobin, trifloxystrobin) showed no increased mortality but only sublethal effects [31]. In contrast, butterfly larvae exposed orally to broad-spectrum fungicides, including difenoconazole, showed additionally increased mortality [24,30]. We here observed sublethal effects on larval and pupal development time as well as on relative fat content. However, these were mainly driven by contrasts between the sulphur-based fungicide Thiovit Jet® and Sercadis® (SDHI fungicide). This might be attributed to differences in the mode of action of these fungicides, as sulphur is generally considered a multi-site contact fungicide with non-specific activity [61], and its antifungal effects have been associated with intracellular redox disturbances and mitochondrial respiration inhibition [62,63], whereas fluxapyroxad (SDHI) specifically inhibits mitochondrial respiration via succinate dehydrogenase [38], which may lead to differences in energy metabolism and growth-related traits. Individuals exposed to Thiovit Jet® as larvae developed slower and had a lower relative fat content than those exposed to Sercadis®, and both sulphur formulations prolonged pupal development relative to Sercadis® and the untreated control. Thus, sulphur-based fungicides induced subtle sublethal negative effects, although they are generally considered to have relatively low ecotoxicity for non-target organisms such as bees and predatory mites [33]. However, assessments often do not capture sublethal effects.

Sublethal effects may have important ecological consequences. Prolonged larval development may indicate compensatory mechanisms to reach a certain body size after suboptimal early conditions (i.e., catch-up growth; [64,65]). However, this comes at a cost of elevated mortality risks [66–68]. This risk might be even more pronounced during the immobile pupal stage [69]. Reduced lipid reserves may impair adult fitness by reducing flight performance, dispersal capacity, and reproductive success [70,71]. Thus, sublethal effects may translate into reduced survival and reproduction in the field, even if survival in the laboratory was unaffected. Such effects could also be relevant for threatened species inhabiting vineyards, such as the Mosel Apollo (*P. apollo vinningensis*), where reductions in individual fitness may further compromise already vulnerable populations. However, empirical data for this species are lacking, and further research is warranted.

Interestingly, fluxapyroxad did not result in any detectable negative effects, although it may in principle impair mitochondrial function in many organisms [38,39]. The absence of detectable oral toxicity of Sercadis® in the present study may reflect (1) limited uptake of the active ingredient from treated leaves, (2) rapid detoxification, and / or (3) concentrations below effect thresholds for *P. rapae*, as insects possess detoxification systems capable of metabolizing xenobiotics [72,73]. Among these, detoxification is supported by previous studies in insects, including *P. rapae* [73,74]. Our results might be specific to the concentrations used and the organism tested, as other studies did show adverse effects of fluxapyroxad on non-target organisms; suggesting species-specific responses complicating transfer across taxa. Fluxapyroxad induced, for instance, developmental abnormalities, oxidative stress and impaired haematopoiesis in zebrafish (*Danio rerio*) embryos [74], and caused histopathological damage and oxidative stress in the earthworm *Eisenia fetida* [42]. Moreover, other SDHI fungicides such as fluopyram have been reported to cause sublethal effects in aquatic organisms [37].

Regarding sexual differences, males developed faster, reached a higher pupal mass and a higher fat content than females. These patterns are well-known in *Pieris* butterflies, being driven by protandry selection and a high investment into spermatophores and flight to secure matings [75,76]. The absence of significant sex by treatment interactions indicates that fungicide exposure did not differentially affect males and females in our study.

Overall, sulphur exposure caused sublethal effects in our study while fluxapyroxad did not. Importantly, though, only one effect was significant relative to controls, which is a prolonged pupal development in both sulphur-based fungicides. However, the documented sublethal effects were only subtle throughout, resulting in a by 1.3 days or 17.3% longer pupal development compared to controls.

### 4.2 Contact exposure: concentration-dependent mortality

In contrast to oral exposure, contact exposure resulted in a clear increase in mortality with no differences among the three fungicides tested. This demonstrates that exposure route might be a critical determinant of toxicity, as cuticular uptake may lead to a more direct disruption of physiological processes than ingestion, particularly for compounds with limited gut absorption [72,77]. Contact exposure may thus be associated with more uniform responses among fungicides, which may be expected given differences in uptake and internal processing between exposure routes. However, the lack of significant differences among fungicides should also be interpreted in the light of the relatively small sample sizes, which may have limited the ability to detect differences among treatments. Nevertheless, all three fungicides consistently caused high mortality under contact exposure, highlighting the strong overall toxicity of this exposure route, which may mask differences among compounds. Our results are in line with some others on arthropods. For instance, Zhang et al. ([41]) reported that fluxapyroxad and other SDHI fungicides caused significant mortality in springtails (*Folsomia candida*) under contact exposure. Contact to sulphur dust reduced egg laying, egg hatching rate, and larval settlement in the non‑target moth *Lobesia botrana* under both laboratory and field conditions [35]. Sulphur applications have also been shown to reduce survival and reproduction in the predatory mites *Neoseiulus californicus* [35] and *Galendromus occidentalis* [34], demonstrating potential impacts on beneficial arthropods.

### 4.3 Contrasting effects of oral and contact exposure on mortality

The contrasting effects of fungicides between oral and contact exposure likely reflect differences in uptake pathways. Contact uptake must first traverse the cuticle, which can limit penetration but once overcome, delivers compounds directly into the haemolymph, bypassing gut barriers [78]. Conversely, orally ingested compounds must cross the midgut epithelium before reaching the haemolymph, where passive and active transport mechanisms as well as detoxification processes can limit systemic exposure, possibly reducing acute toxicity [72]. These distinct uptake pathways likely contribute to the higher mortality observed in our contact exposure experiment compared to oral exposure. Note that mortality was already substantially elevated at the lowest contact concentration, suggesting that uptake route might be a critical determinant of toxicity, with contact being potentially more detrimental than ingestion.

### 4.4 Conclusions

Our results demonstrate that fungicides can affect Lepidoptera in a route-dependent manner, with subtle but significant sublethal effects on development and energy reserves after oral exposure, and a substantial increase in mortality after contact exposure. Interestingly, differences among fungicides were mainly negligible. However, the tested fungicides differ largely in environmental persistence, which was not considered in our experiments. Elemental sulphur oxidizes rapidly in vineyard soils, mainly within 30 minutes, and complete oxidation occurs within seven days [79]. In contrast, fluxapyroxad is highly persistent in soils, with residues being detectable for up to 183 days [39,80,81]. Consequently, the long environmental persistence of fluxapyroxad may result in chronic exposure and interactions with other pesticides, such that its toxicity may be underestimated in short-term laboratory experiments.

Our results suggest that direct contact to fungicides comprises a severe threat to insect communities of vineyards. Therefore, management strategies that reduce larval contact with spray droplets may substantially mitigate non-target effects. Such measures may include targeted application techniques (e.g., drone-based instead of helicopter spraying) and scheduling applications to time periods when larvae are less active or retreat into shelter (e.g., during night). In addition to a careful selection of products, such mitigation strategies may greatly benefit insect communities of vineyards including non-target Lepidoptera. Our findings highlight the need for further studies, as fungicides may exert substantial lethal and sublethal effects on Lepidoptera.

## Supporting information

S1 Table Coordinates, capture date, and butterfly IDs of fecund *Pieris rapae* females caught for oviposition in July (oral exposure) and August (contact exposure) 2025. (DOCX)

S2 Table Calibration of fungicide applications for oral exposure. (DOCX)

S3 Table Overview and calculation of fungicide application rates for oral exposure experiments. (DOCX)

S4 Table Fungicide treatments and concentrations used for contact exposure. (DOCX)

S5 Table Results of linear mixed-effects models for fungicide oral exposure in *Pieris rapae.* (DOCX)

S6 Table Effects of treatment on various traits in *Pieris rapae* after oral exposure. (DOCX)

S7 Table Effects of treatment and sex on various traits in *Pieris rapae* after oral exposure. (DOCX)

S8 Table Survival of *Pieris rapae* after contact exposure to fungicides at different concentrations. (DOCX)

S9 Table Model-based effect sizes and 95% confidence intervals for various traits in *Pieris rapae* following oral exposure. (DOCX)

S10 Table Model-based effect sizes and 95% confidence intervals for mortality in *Pieris rapae* following contact exposure. (DOCX)

S1 Fig Experimental setup for oral exposure and fungicide spraying. (DOCX)

S2 Fig Calibration of the hand sprayer for oral fungicide exposure. (DOCX)

S3 Fig Experimental setup for contact exposure. (DOCX)

S4 Fig Calibration of fungicide contact exposure. (DOCX)

S1 Text Calibration of the hand sprayer for oral exposure. (DOCX)

S2 Text Calibration of the hand sprayer and calculation of applied fungicide amounts for contact exposure. (DOCX)

S1 Data Dataset on mortality and sublethal effects of sulphur-based and fluxapyroxad fungicides in *Pieris rapae.* (DOCX)
