# Supplementary material for: Exposure route mediates toxicological effects of sulphur and fluxapyroxad fungicides in a non-target butterfly
Source: PLoS One. 2026 Jul 9;21(7):e0353528. doi: 10.1371/journal.pone.0353528 (PMC13349104; doi:10.1371/journal.pone.0353528)
Supplement: S1 Table — (DOCX) [file pone.0353528.s001.docx]

**S1 Table. Coordinates, capture date, and butterfly IDs of fecund *Pieris rapae* females caught for oviposition in July (oral exposure) and August (contact exposure) 2025.**

| **Oral exposure**  **(11 & 19 July 2025)** | | **Contact exposure**  **(13 August 2025)** | |
| --- | --- | --- | --- |
| **Mother ID** | **Coordinates** | **Mother ID** | **Coordinates** |
| J1 | 50°16′24.8″ N, 7°19′51,3″ E | A1 | 50°16′34″N 7°17′36.3″E |
| J2 | 50°16′24.8″ N, 7°19′51,3″ E | A2 | 50°16′34″N 7°17′36.3″E |
| J3 | 50°16′26″ N, 7°17′49″ E | A3 | 50°16′34″N 7°17′36.3″E |
| J4 | 50°16′26″ N, 7°17′49″ E | A4 | 50°16′34″N 7°17′36.3″E |
| J5 | 50°16′26″ N, 7°17′49″ E | A5 | 50°16′34″N 7°17′36.3″E |
| J6 | 50°16′26″ N, 7°17′49″ E | A6 | 50°16′34″N 7°17′36.3″E |
| J7 | 50°16′26″ N, 7°17′49″ E | A7 | 50°16′24.8″N 7°19′51.3″E |
| J8 | 50°16′26″ N, 7°17′49″ E | A8 | 50°17'38.9"N 7°19'15.2"E |
| J9 | 50°16′26″ N, 7°17′49″ E |  |  |
| J10 | 50°16′24.8″ N, 7°19′51,3″ E |  |  |
| J11 | 50°16′26″ N, 7°17′49″ E |  |  |
| J12 | 50°16′26″ N, 7°17′49″ E |  |  |
| J13 | 50°16′26″ N, 7°17′49″ E |  |  |
| J14 | 50°16′26″ N, 7°17′49″ E |  |  |
| J15 | 50°16′26″ N, 7°17′49″ E |  |  |
| J16 | 50°21'52.5"N 7°33'34.1"E |  |  |
| J17 | 50°21'45.7"N 7°33'25.1"E |  |  |

Females were sampled using sweep netting in field margins of rapeseed fields and urban habitats such as hedgerows and embankments.
