# Supplementary material for: Exposure route mediates toxicological effects of sulphur and fluxapyroxad fungicides in a non-target butterfly
Source: PLoS One. 2026 Jul 9;21(7):e0353528. doi: 10.1371/journal.pone.0353528 (PMC13349104; doi:10.1371/journal.pone.0353528)
Supplement: S2 Table — (DOCX) [file pone.0353528.s002.docx]

**S2 Table.** **Calibration of fungicide applications for oral exposure.**

| **Petri Dish ID** | **Treatment** | **Initial Weight** | **24 h after spraying** | **48 h after spraying** | **Δ 24 h** | **Δ 48 h** |
| --- | --- | --- | --- | --- | --- | --- |
| S1 | Stulln® | 564.0 | 575.1 | 573.4 | 11.1 | 9.4 |
| S2 | Stulln® | 563.7 | 573.4 | 572.7 | 9.7 | 9.0 |
| S3 | Stulln® | 568.8 | 579.3 | 578.3 | 10.5 | 9.5 |
| S4 | Stulln® | 579.5 | 590.0 | 588.6 | 10.5 | 9.1 |
| S5 | Stulln® | 553.0 | 563.1 | 561.9 | 10.1 | 8.9 |
| S6 | Stulln® | 572.5 | 582.5 | 581.6 | 10.0 | 9.1 |
| Se1 | Sercadis® | 568.4 | 578.3 | 577.9 | 9.9 | 9.5 |
| Se2 | Sercadis® | 590.9 | 600.5 | 599.9 | 9.6 | 9.0 |
| Se3 | Sercadis® | 614.2 | 624.4 | 623.0 | 10.2 | 8.8 |
| Se4 | Sercadis® | 583.1 | 592.8 | 591.7 | 9.7 | 8.6 |
| Se5 | Sercadis® | 585.1 | 594.5 | 593.5 | 9.4 | 8.4 |
| Se6 | Sercadis® | 585.2 | 594.7 | 593.5 | 9.5 | 8.3 |
| T1 | Thiovit Jet® | 579.5 | 590.2 | 590.0 | 10.7 | 10.5 |
| T2 | Thiovit Jet® | 601.1 | 612.4 | 611.5 | 11.3 | 10.4 |
| T3 | Thiovit Jet® | 566.4 | 577.4 | 575.8 | 11.0 | 9.4 |
| T4 | Thiovit Jet® | 562.7 | 573.0 | 571.8 | 10.3 | 9.1 |
| T5 | Thiovit Jet® | 601.0 | 612.3 | 611.3 | 11.3 | 10.3 |
| T6 | Thiovit Jet® | 572.4 | 583.1 | 581.7 | 10.7 | 9.3 |

Calibration of fungicide applications for oral exposure. Given are petri dish IDs, treatment, and filter paper weights in milligrams (mg). Initial weight = weight of the filter paper before spraying. 24 h after spraying = weight after drying under a fume hood for 24 h. 48 h after spraying = weight after drying under a fume hood for 48 h. Δ 24 h = weight after 24 h minus initial weight; Δ 48 h = weight after 48 h minus initial weight. Positive values indicate increased weight due to spraying. Filter papers were used to calibrate the spray bottles, with 100 ml of the respective fungicide solution applied per 1 m² within 12 seconds (SM1, Fig. S2). The values show consistent spray deposition across all treatments, with mean ± SE values of 10.3 ± 0.2 mg for Stulln®, 9.7 ± 0.1 mg for Sercadis®, and 10.9 ± 0.2 mg for Thiovit Jet® 24 h after spraying, and 9.2 ± 0.1 mg, 8.8 ± 0.2 mg, and 9.8 ± 0.3 mg 48 h after spraying, respectively, indicating that the spray bottles delivered uniform amounts and that the spraying was applied evenly.
