# Supplementary material for: Exposure route mediates toxicological effects of sulphur and fluxapyroxad fungicides in a non-target butterfly
Source: PLoS One. 2026 Jul 9;21(7):e0353528. doi: 10.1371/journal.pone.0353528 (PMC13349104; doi:10.1371/journal.pone.0353528)
Supplement: S3 Table — (DOCX) [file pone.0353528.s003.docx]

**S3 Table. Overview and calculation of fungicide application rates for oral exposure experiments.**

| **Fungicide** | **Applied helicopter dose** | **Applied dose (m² / 100 ml)** | **Dose at ground level (m² / 100 ml)** | **% of applied dose** | **Active ingredient (µg/cm² leaf area)** |
| --- | --- | --- | --- | --- | --- |
| **Stulln®** | 4.000 kg/ha | 0.400 g | 0.197 g | 49.2 | 15.76 |
| **Sercadis®** | 0.120 L/ha | 0.012 ml | 0.005 g | 45.3 | 0.15 |
| **Thiovit Jet®** | 4.000 kg/ha | 0.400 g | 0.198 g | 49.4 | 15.84 |

Overview of fungicide application for oral exposure. Applied doses per hectare were derived from helicopter spray data (applied helicopter dose) and recalculated to a per-square-metre basis (applied dose; m² / 100 ml). Doses at ground level (m² / 100 ml) in the current study were determined gravimetrically using six petri dishes per treatment plot (S1B Fig.). The values represent the amount of spray solution (formulated product) deposited per square metre, and the percentage shows the ratio of actual to ground level dose. Active ingredient concentrations (µg/cm² leaf area) were derived from gravimetrically measured deposition (dose at ground level) and product-specific active ingredient concentrations (800 g/kg elemental sulphur for both sulphur fungicides, and 300 g/L fluxapyroxad in Sercadis ®) and expressed per cm² via unit conversion.
