# Supplementary material for: Exposure route mediates toxicological effects of sulphur and fluxapyroxad fungicides in a non-target butterfly
Source: PLoS One. 2026 Jul 9;21(7):e0353528. doi: 10.1371/journal.pone.0353528 (PMC13349104; doi:10.1371/journal.pone.0353528)
Supplement: S4 Table — (DOCX) [file pone.0353528.s004.docx]

**S4 Table. Fungicide treatments and concentrations used for contact exposure.**

| **Fungicide** | **Max. field dose** | **100** | **33.3** | **11.1** | **3.7** | **1.23** | **0.41** | **0.0457** |
| --- | --- | --- | --- | --- | --- | --- | --- | --- |
| Stulln® (concentration) | 8kg/ha in 1000L water | 80.000 g/L | 26.670 g/L | 8.900 g/L | 2.970 g/L | 0.990 g/L | 0.330 g/L | 0.037 g/L |
| Stulln®  (P applied) |  | 14.304 mg | 3.039 mg | 0.839 mg | 0.426 mg | 0.115 mg | 0.060 mg | 0.006 mg |
| Stulln®  (AI applied) |  | 91.546 µg | 19.450 µg | 5.370 µg | 2.726 µg | 0.726 µg | 0.384 µg | 0.038 µg |
| Thiovit Jet® (concentration) | 8kg/ha in 1000L water | 80.000 g/L | 26.670 g/L | 8.900 g/L | 2.970 g/L | 0.990 g/L | 0.330 g/L | 0.037 g/L |
| Thiovit Jet®  (P applied) |  | 21.691 mg | 8.699 mg | 3.375 mg | 1.220 mg | 0.412 mg | 0.157 mg | 0.016 mg |
| Thiovit Jet® (AI applied) |  | 138.822 µg | 55.674 µg | 21.600 µg | 7.808 µg | 2.637 µg | 1.005 µg | 0.237 µg |
| Sercadis® (concentration) | 0.24L/ha in 150L water | 16.000 ml/L | 5.300 ml/L | 1.800 ml/L | 0.590 ml/L | 0.200 ml/L | 0.070 ml/L | 0.007 ml/L |
| Sercadis®  (P applied) |  | 4.055 mg | 2.614 mg | 0.435 mg | 0.112 mg | 0.051 mg | 0.016 mg | 0.0016 mg |
| Sercadis®  (AI applied) |  | 9.732 µg | 6.274 µg | 1.044 µg | 0.269 µg | 0.122 µg | 0.038 µg | 0.004 µg |

Fungicide treatments and concentrations used for contact exposure, including a stock solution corresponding to 100 % (defined as 10x the maximum recommended field concentration) and subsequent dilutions (33.3 %, 11.1 %, 3.7 %, 1.23 %, 0.41 %, 0.04 %) plus the control (0 %; only water, no active ingredient). For each fungicide treatment, the concentration in the spray solution and the actual amount of formulated product (P) applied onto the pre-marked exposure area (10 cm x 12.5 cm; S3 Fig) are reported. Active ingredient applied (AI) per cm^2^ spray area (µg/cm^2^) was calculated from the actual amount of formulated product (P) applied per spray area (125 cm^2^) and the respective active ingredient content (c; 800 g/kg elemental sulphur for both sulphur fungicides, and 300 g/L fluxapyroxad in Sercadis ®). AI applied was calculated as: AI applied = (P applied × c × 1000) / 125; expressed in µg (×1000 conversion from mg).
