# Supplementary material for: Exposure route mediates toxicological effects of sulphur and fluxapyroxad fungicides in a non-target butterfly
Source: PLoS One. 2026 Jul 9;21(7):e0353528. doi: 10.1371/journal.pone.0353528 (PMC13349104; doi:10.1371/journal.pone.0353528)
Supplement: S5 Table — (DOCX) [file pone.0353528.s005.docx]

**S5 Table**. **Results of linear mixed-effects models for fungicide oral exposure in Pieris rapae.**

| **Larval time** | **df** | **MS** | **F** | **p** |
| --- | --- | --- | --- | --- |
| Treatment | 3, 194 | 0.04 | 3.46 | **0.018** |
| Sex | 1, 196 | 0.03 | 2.40 | 0.123 |
| T x Sex | 3, 193 | 0.02 | 1.62 | 0.187 |
| **Pupal time** | **df** | **MS** | **F** | **p** |
| Treatment | 3, 195 | 0.54 | 83.39 | **< 0.001** |
| Sex | 1, 198 | 0.03 | 4.86 | **0.029** |
| T x Sex | 3, 194 | < 0.01 | 1.48 | 0.223 |
| **Pupal mass** | **df** | **MS** | **F** | **p** |
| Treatment | 3, 192 | 62.76 | 0.37 | 0.775 |
| Sex | 1, 193 | 1490.73 | 8.78 | **0.003** |
| T x Sex | 3, 191 | 69.63 | 0.41 | 0.746 |
| **Growth rate** | **df** | **MS** | **F** | **p** |
| Treatment | 3, 191 | < 0.01 | 2.45 | 0.065 |
| Sex | 1, 194 | < 0.01 | 0.85 | 0.359 |
| T x Sex | 3, 190 | < 0.01 | 1.39 | 0.247 |
| **Thorax mass** | **df** | **MS** | **F** | **p** |
| Treatment | 3, 196 | 0.07 | 1.40 | 0.243 |
| Sex | 1, 199 | 0.04 | 0.73 | 0.395 |
| T x Sex | 3, 195 | 0.01 | 0.08 | 0.972 |
| **Abdomen mass** | **df** | **MS** | **F** | **p** |
| Treatment | 3, 174 | 5.94 | 0.40 | 0.752 |
| Sex | 1, 182 | 3.28 | 0.22 | 0.638 |
| T x Sex | 3, 174 | 7.30 | 0.49 | 0.687 |

S5 Table continued

| **TA ratio** | **df** | **MS** | **F** | **p** |
| --- | --- | --- | --- | --- |
| Treatment | 3,178 | 0.16 | 2.19 | 0.091 |
| Sex | 1,181 | 0.09 | 1.17 | 0.280 |
| T x Sex | 3,178 | 0.03 | 0.35 | 0.793 |
| **Wing length** | **Df** | **MS** | **F** | **p** |
| Treatment | 3,191 | 0.04 | 2.01 | 0.114 |
| Sex | 1,195 | 0.04 | 2.13 | 0.146 |
| T x Sex | 3,190 | 0.01 | 0.65 | 0.581 |
| **Relative fat** | **Df** | **MS** | **F** | **p** |
| Treatment | 3,177 | 144.01 | 3.78 | **0.012** |
| Sex | 1,179 | 151.91 | 3.98 | **0.047** |
| T x Sex | 3, 177 | 76.56 | 2.01 | 0.115 |

Results of linear mixed-effects models (Type III ANOVA with Satterthwaite’s method) for the effects of fungicide oral exposure (treatment) on *Pieris rapae*. Shown are the effects of treatment, sex, and their interaction on various traits. T: treatment, TA ratio: thorax-abdomen ratio. Only surviving individuals were considered for scoring the traits below. Significant p-values are given bold. Sample sizes per trait and treatment group are reported in S6 and S7 Tables. Model-based effect sizes and 95% confidence intervals are provided in S9 Table.
