# Supplementary material for: Exposure route mediates toxicological effects of sulphur and fluxapyroxad fungicides in a non-target butterfly
Source: PLoS One. 2026 Jul 9;21(7):e0353528. doi: 10.1371/journal.pone.0353528 (PMC13349104; doi:10.1371/journal.pone.0353528)
Supplement: S6 Table — (DOCX) [file pone.0353528.s006.docx]

**S6 Table. Effects of treatment on various traits in Pieris rapae after oral exposure.**

| **Trait** | **Control** | **Stulln**® | **Sercadis**® | **Thiovit Jet**® |
| --- | --- | --- | --- | --- |
| **Larval time** | 14.8 ± 0.4_(ab)_  (n = 50) | 14.7 ± 0.4_(ab)_  (n = 46) | 14.2 ± 0.4_(a)_  (n = 54) | 15.2 ± 0.4_(b)_  (n = 60) |
| **Pupal time** | 7.5 ± 0.1_(a)_  (n = 50) | 8.8 ± 0.2_(b)_  (n = 46) | 7.2 ± 0.1_(c)_  (n = 54) | 8.8 ± 0.2_(b)_  (n = 60) |
| **Pupal mass** | 123.2 ± 3.5  (n = 49) | 124.5 ± 3.7  (n = 46) | 122.3 ± 3.3  (n = 53) | 124.8 ± 3.1  (n = 60) |
| **Growth rate** | 0.3 ± 0.1  (n = 49) | 0.3 ± 0.1  (n = 46) | 0.3 ± 0.1  (n = 53) | 0.3 ± 0.1  (n = 60) |
| **Thorax mass** | 5.1 ± 0.3  (n = 50) | 4.7 ± 0.3  (n = 46) | 4.8 ± 0.3  (n = 54) | 4.9 ± 0.2  (n = 60) |
| **Abdomen mass** | 17.9 ± 0.9  (n = 46) | 18.7 ± 1.0  (n = 41) | 18.4 ± 0.9  (n = 51) | 18.6 ± 0.8  (n = 56) |
| **TA ratio** | 0.3 ± < 0.1  (n = 46) | 0.3 ± < 0.1  (n = 41) | 0.3 ± < 0.1  (n = 51) | 0.3 ± < 0.1  (n = 56) |
| **Wing length** | 23.3 ± 0.7  (n = 50) | 22.5 ± 0.8  (n = 43) | 24.0 ± 0.8  (n = 54) | 24.0 ± 0.7  (n = 59) |
| **Relative fat** | 47.2 ± 1.6_(ab)_  (n = 46) | 47.2 ± 1.8_(ab)_  (n = 41) | 48.9 ± 1.6_(a)_  (n = 51) | 44.8 ± 1.5_(b)_  (n = 56) |

Given are means ± 1 SE, and sample sizes (n) per treatment; only surviving individuals were considered for scoring these traits. TA ratio: thorax-abdomen ratio. Different subscript letters in parentheses within rows indicate significant post hoc (Tukey) differences across treatments. No letters indicate no overall significant treatment effect, such that no post hoc was performed.
