# Supplementary material for: Exposure route mediates toxicological effects of sulphur and fluxapyroxad fungicides in a non-target butterfly
Source: PLoS One. 2026 Jul 9;21(7):e0353528. doi: 10.1371/journal.pone.0353528 (PMC13349104; doi:10.1371/journal.pone.0353528)
Supplement: S7 Table — (DOCX) [file pone.0353528.s007.docx]

**S7 Table. Effects of treatment and sex on traits in Pieris rapae after oral exposure.**

|  | **Control** | | **Stulln**® | | **Sercadis**® | | **Thiovit Jet**® | |
| --- | --- | --- | --- | --- | --- | --- | --- | --- |
| **Trait** | **Male** | **Female** | **Male** | **Female** | **Male** | **Female** | **Male** | **Female** |
| **Larval time** | 15.3 ± 0.4  (n = 28) | 14.2 ± 0.4  (n = 22) | 15.0 ± 0.5  (n = 13) | 14.5 ± 0.3  (n = 33) | 14.1 ± 0.3  (n = 34) | 14.2 ± 0.4  (n = 20) | 15.1 ± 0.4  (n = 30) | 15.2 ± 0.4  (n = 30) |
| **Pupal time** | 7.3 ± 0.1  (n = 28) | 7.7 ± 0.2  (n = 22) | 8.7 ± 0.2  (n = 13) | 8.9 ± 0.2  (n = 33) | 7.2 ± 0.1  (n = 34) | 7.1 ± 0.2  (n = 20) | 8.7 ± 0.2  (n = 30) | 8.9 ± 0.2  (n = 30) |
| **Pupal mass** | 127.8 ± 3.1  (n = 28) | 118.6 ± 3.6  (n = 21) | 126.6 ± 4.2  (n = 13) | 122.3 ± 3.1  (n = 33) | 125.2 ± 3.0  (n = 33) | 119.4 ± 3.6  (n = 20) | 126.7 ± 3.1  (n = 30) | 122.8 ± 3.2  (n = 30) |
| **Growth rate** | 0.3 ± 0.1  (n = 28) | 0.3 ± 0.1  (n = 21) | 0.3 ± 0.1  (n = 13) | 0.3 ± 0.1  (n = 33) | 0.3 ± 0.1  (n = 33) | 0.3 ± 0.1  (n = 20) | 0.3 ± 0.1  (n = 30) | 0.3 ± 0.1  (n = 30) |
| **Thorax mass** | 5.3 ± 0.3  (n = 28) | 5.1 ± 0.3  (n = 22) | 4.7 ± 0.3  (n = 13) | 4.7 ± 0.2  (n = 33) | 4.9 ± 0.2  (n = 34) | 4.7 ± 0.3  (n = 20) | 4.9 ± 0.2  (n = 30) | 4.8 ± 0.2  (n = 30) |
| **Abdomen mass** | 18.0 ± 0.8  (n = 25) | 17.7 ± 0.9  (n = 21) | 18.3 ± 1.2  (n = 12) | 19.1 ± 0.8  (n = 29) | 18.6 ± 0.8  (n = 31) | 18.2 ± 1.0  (n = 20) | 18.1 ± 0.8  (n = 26) | 19.2 ± 0.8  (n = 30) |
| **TA ratio** | 0.3 ± < 0.1  (n = 25) | 0.3 ± < 0.1  (n = 21) | 0.3 ± < 0.1  (n = 12) | 0.3 ± < 0.1  (n = 29) | 0.3 ± < 0.1  (n = 31) | 0.3 ± < 0.1  (n = 20) | 0.3 ± < 0.1  (n = 26) | 0.3 ± < 0.1  (n = 30) |
| **Wing length** | 23.2 ± 0.7  (n = 28) | 23.4 ± 0.8  (n = 22) | 21.7 ± 0.9  (n = 12) | 23.3 ± 0.7  (n = 31) | 23.5 ± 0.6  (n = 34) | 24.5 ± 0.9  (n = 20) | 24.0 ± 0.7  (n = 29) | 24.0 ± 0.7  (n = 30) |
| **Relative fat** | 49.5 ± 1.5  (n = 25) | 44.9 ± 1.6  (n = 21) | 49.0 ± 2.0  (n = 12) | 45.4 ± 1.5  (n = 29) | 48.8 ± 1.4  (n = 31) | 49.0 ± 1.7  (n = 20) | 44.6 ± 1.5  (n = 26) | 45.0 ± 1.4  (n = 30) |

Given are means ± 1 SE, and sample sizes (n) per treatment and sex.
