# Supplementary material for: Exposure route mediates toxicological effects of sulphur and fluxapyroxad fungicides in a non-target butterfly
Source: PLoS One. 2026 Jul 9;21(7):e0353528. doi: 10.1371/journal.pone.0353528 (PMC13349104; doi:10.1371/journal.pone.0353528)
Supplement: S8 Table — (DOCX) [file pone.0353528.s008.docx]

**S8 Table. Survival of *Pieris rapae* after contact exposure to fungicides at different concentrations.**

| **Treatment** | **0.0** | **0.1** | **0.4** | **1.2** | **3.7** | **11.0** | **33.0** | **100.0** |
| --- | --- | --- | --- | --- | --- | --- | --- | --- |
| Stulln® | 90 | 40 | 70 | 60 | 60 | 70 | 60 | 50 |
| Sercadis® | 80 | 30 | 40 | 60 | 30 | 20 | 40 | 60 |
| Thiovit Jet® | 80 | 70 | 70 | 70 | 30 | 80 | 50 | 20 |

Survival (%) of *Pieris rapae* after contact exposure to three fungicides (Stulln®, Sercadis®, Thiovit Jet®) at different concentrations (8 concentration levels per fungicide). 0 % indicates the untreated control, and the treated concentrations (> 0 %) range from 0.1 % to 100 %, with 100 % corresponding to 10x the recommended field rate; the other concentrations are relative percentages of this maximum. Survival is given as percentage; n = 10 individuals per treatment and concentration (total n = 240).
