# Supplementary material for: Exposure route mediates toxicological effects of sulphur and fluxapyroxad fungicides in a non-target butterfly
Source: PLoS One. 2026 Jul 9;21(7):e0353528. doi: 10.1371/journal.pone.0353528 (PMC13349104; doi:10.1371/journal.pone.0353528)
Supplement: S9 Table — (DOCX) [file pone.0353528.s009.docx]

**S9 Table. Model-based effect sizes and 95% confidence intervals for various traits in *Pieris rapae* following oral exposure.**

| **Trait** | **Comparison** | **Effect size** | **CI low** | **CI high** |
| --- | --- | --- | --- | --- |
| Larval time (days) | S – C | -0.01 | -0.06 | 0.05 |
|  | Se – C | -0.04 | -0.09 | 0.01 |
|  | T – C | 0.03 | -0.02 | 0.08 |
| Pupal time (days) | S – C | 0.16 | 0.12 | 0.20 |
|  | Se – C | -0.05 | -0.08 | -0.01 |
|  | T – C | 0.16 | 0.12 | 0.19 |
| Pupal mass (mg) | S – C | 1.25 | -5.69 | 8.18 |
|  | Se – C | -0.91 | -7.31 | 5.49 |
|  | T – C | 1.53 | -4.60 | 7.67 |
| Growth rate (mg/day) | S – C | < -0.01 | -0.02 | 0.02 |
|  | Se – C | 0.01 | -0.01 | 0.03 |
|  | T – C | -0.01 | -0.03 | 0.01 |
| Thorax mass (mg) | S – C | -0.10 | -0.21 | 0.02 |
|  | Se – C | -0.07 | -0.18 | 0.04 |
|  | T – C | -0.06 | -0.16 | 0.05 |
| Abdomen mass (mg) | S – C | 0.81 | -1.29 | 2.91 |
|  | Se – C | 0.53 | -1.38 | 2.44 |
|  | T – C | 0.75 | -1.10 | 2.60 |
| TA ratio | S – C | -0.15 | -0.29 | < 0.01 |
|  | Se – C | -0.11 | -0.25 | 0.02 |
|  | T – C | -0.10 | -0.23 | 0.03 |
| Wing length (mm) | S – C | -0.04 | -0.11 | 0.04 |
|  | Se – C | 0.03 | -0.04 | 0.10 |
|  | T – C | 0.03 | -0.04 | 0.09 |
| Relative fat (%) | S – C | < 0.01 | -3.39 | 3.39 |
|  | Se – C | 1.69 | -1.39 | 4.77 |
|  | T – C | -2.40 | -5.40 | 0.60 |

Estimated treatment effects (S: Stulln®, Se: Sercadis®, T: Thiovit Jet® vs. C: control) on various traits after oral exposure experiment in *P. rapae*. Given are effect sizes with 95% confidence intervals (CI). Effect sizes represent model-based estimated differences in marginal means (treatment vs control). TA ratio: thorax-abdomen ratio.
