# Supplementary material for: Exposure route mediates toxicological effects of sulphur and fluxapyroxad fungicides in a non-target butterfly
Source: PLoS One. 2026 Jul 9;21(7):e0353528. doi: 10.1371/journal.pone.0353528 (PMC13349104; doi:10.1371/journal.pone.0353528)
Supplement: S10 Table — (DOCX) [file pone.0353528.s010.docx]

**S10 Table.** **Model-based effect sizes and 95% confidence intervals for mortality in *Pieris rapae* following contact exposure.**

| **Fixed effect** | **Estimate** | **SE** | **CI low** | **CI high** |
| --- | --- | --- | --- | --- |
| Intercept (reference: Treatment T) | -0.43 | 0.37 | -1.15 | 0.29 |
| Treatment S vs T | -0.06 | 0.39 | -0.83 | 0.72 |
| Treatment Se vs T | 0.64 | 0.40 | -0.14 | 1.42 |
| Concentration | 0.02 | 0.01 | < 0.01 | 0.04 |
| S × Concentration | -0.02 | 0.01 | -0.04 | 0.01 |
| Se × Concentration | -0.03 | 0.01 | -0.05 | < 0.01 |

Estimated fixed effects (S: Stulln®, Se: Sercadis®, T: Thiovit Jet®) from a generalized linear mixed model with binomial error distribution and logit link function. Given are effect sizes, standard errors (SE), and 95% confidence intervals (CI). Effects represent model-based estimates for treatment, concentration, and their interaction, with T as the reference treatment.
