# Supplementary material for: Exposure route mediates toxicological effects of sulphur and fluxapyroxad fungicides in a non-target butterfly
Source: PLoS One. 2026 Jul 9;21(7):e0353528. doi: 10.1371/journal.pone.0353528 (PMC13349104; doi:10.1371/journal.pone.0353528)
Supplement: S1 Fig — (DOCX) [file pone.0353528.s011.docx]

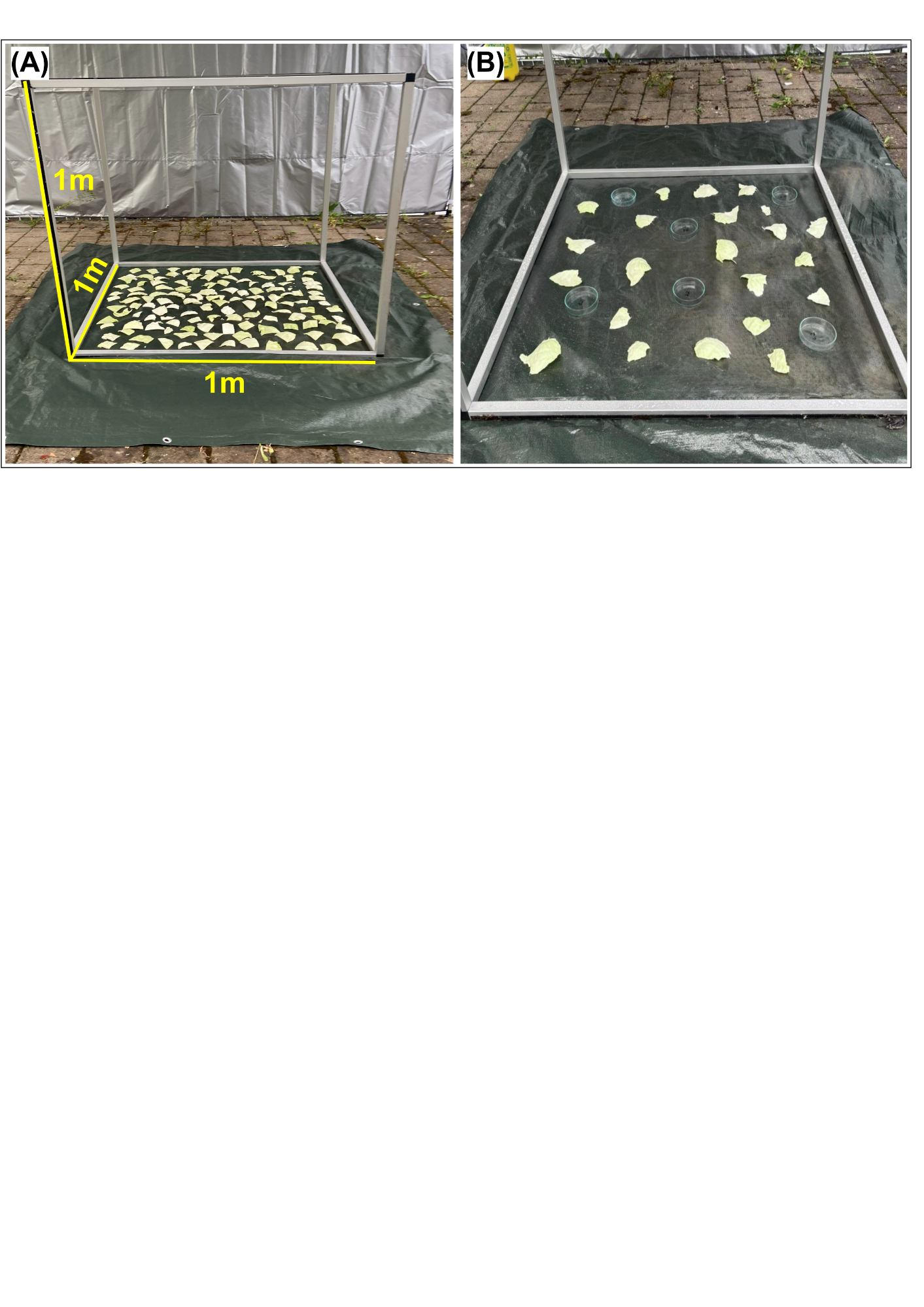


**S1 Fig. Experimental setup for oral exposure and fungicide spraying.**

Experimental setup for the oral exposure of fungicide-treated cabbage leaves: (A) for fungicide spraying leaves were arranged within a 1 m² plot inside a 1 m³ cage, ensuring a 1 m spraying height; (B) six petri dishes were placed randomly in the cage to determine the actual amount of fungicide deposited per m².
