# Supplementary material for: Exposure route mediates toxicological effects of sulphur and fluxapyroxad fungicides in a non-target butterfly
Source: PLoS One. 2026 Jul 9;21(7):e0353528. doi: 10.1371/journal.pone.0353528 (PMC13349104; doi:10.1371/journal.pone.0353528)
Supplement: S2 Fig — (DOCX) [file pone.0353528.s012.docx]

**
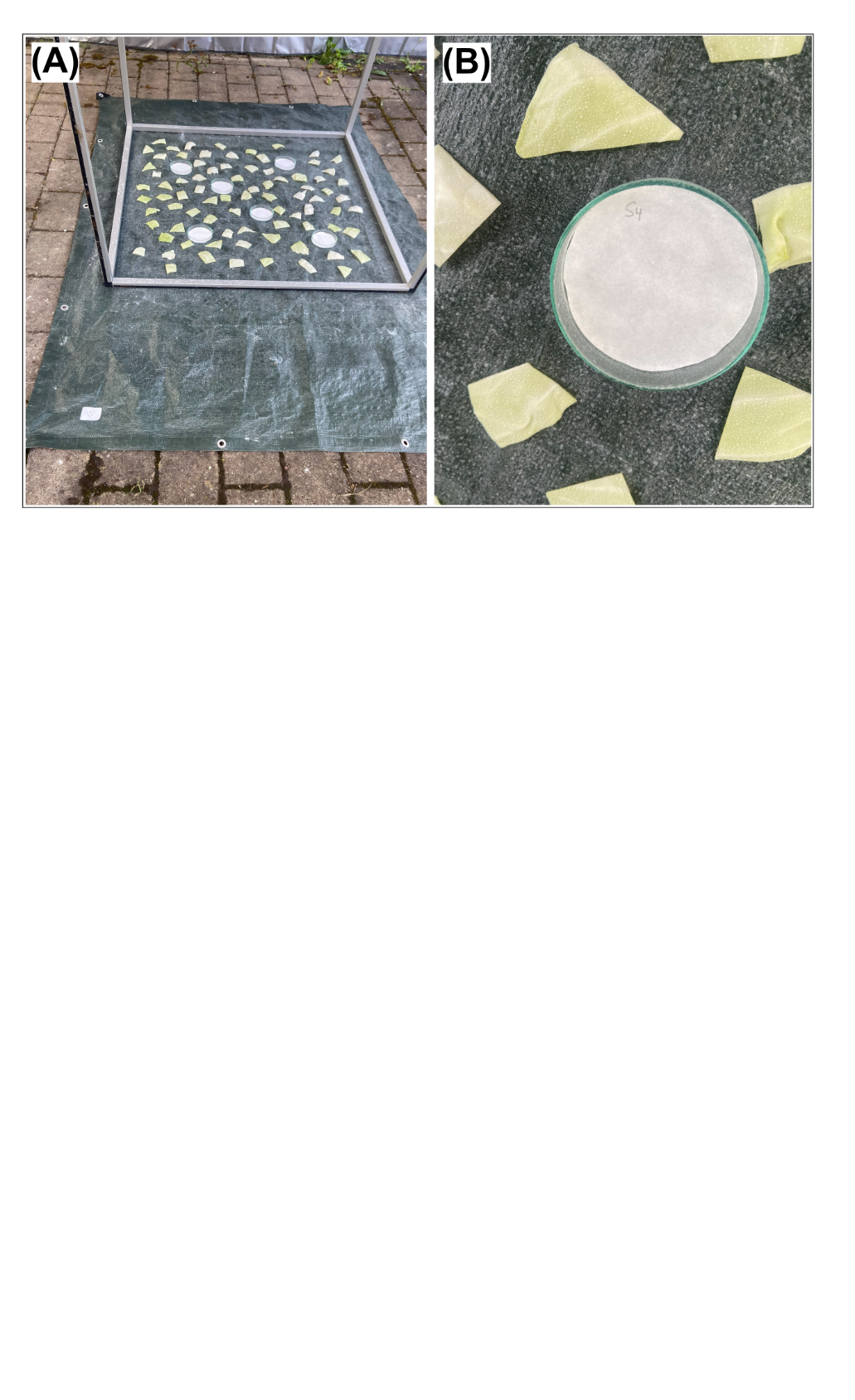
**

**S2 Fig. Calibration of the hand sprayer for oral fungicide exposure.**

For the calibration of the hand sprayer for oral fungicide exposure, cabbage leaves were arranged on a 1 m² spraying area to monitor spray coverage, and six petri dishes with filter paper were randomly placed to verify deposition. Stulln® (S) is shown as an example for both (A) the overview of the spraying area and (B) the close-up of a petri dish and cabbage leaves illustrating uniform fungicide coverage.
