# Supplementary material for: Exposure route mediates toxicological effects of sulphur and fluxapyroxad fungicides in a non-target butterfly
Source: PLoS One. 2026 Jul 9;21(7):e0353528. doi: 10.1371/journal.pone.0353528 (PMC13349104; doi:10.1371/journal.pone.0353528)
Supplement: S3 Fig — (DOCX) [file pone.0353528.s013.docx]

**
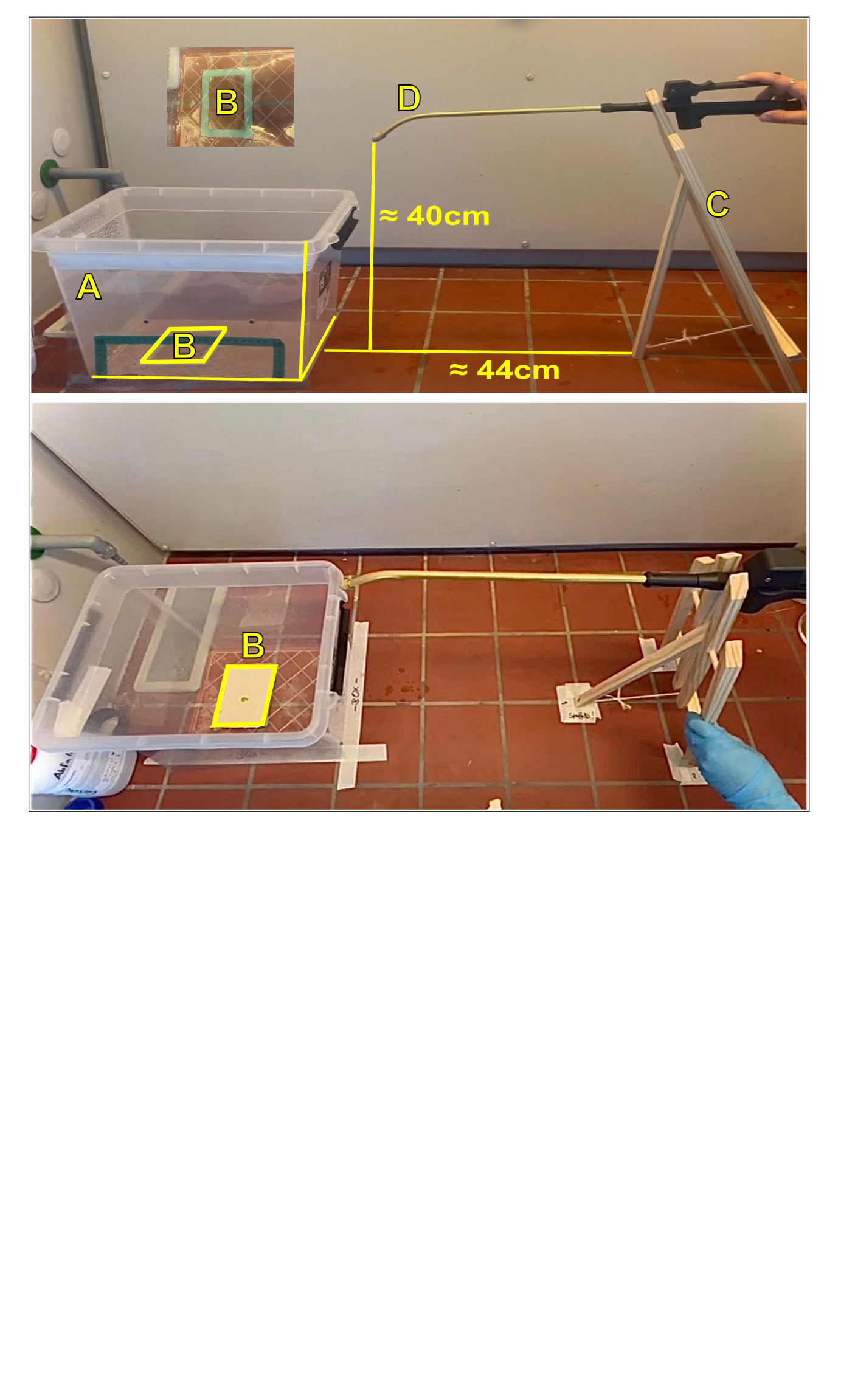
**

**S3 Fig.** **Experimental setup for contact exposure.**

**Experimental setup for contact exposure under a fume hood:** (A) Spraying box measuring 39 cm x 29 cm x 26 cm (width x length x height), with (B) a predefined exposure area for the larva on cellulose paper, measuring 10 cm x 12.5 cm, (C) an easel used to fix the sprayer position and height controlling the distance of the nozzle of the Gloria 3 hand sprayer (D) to the target area for uniform application.
