# Supplementary material for: Exposure route mediates toxicological effects of sulphur and fluxapyroxad fungicides in a non-target butterfly
Source: PLoS One. 2026 Jul 9;21(7):e0353528. doi: 10.1371/journal.pone.0353528 (PMC13349104; doi:10.1371/journal.pone.0353528)
Supplement: S4 Fig — (DOCX) [file pone.0353528.s014.docx]

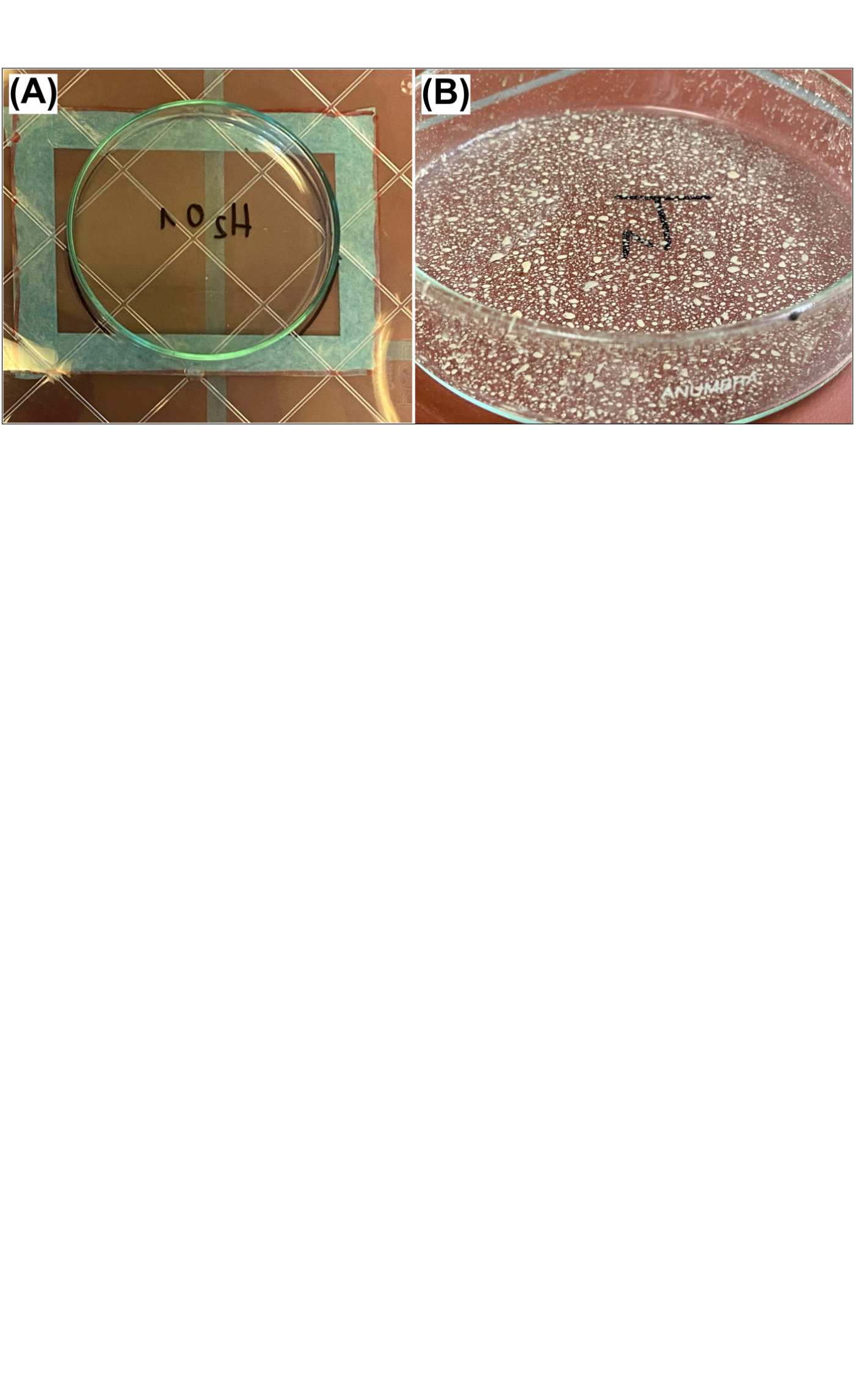


**S4 Fig. Calibration of fungicide contact exposure.**

Calibration of fungicide contact exposure, (A) showing the position of the petri dishes (10 cm x 12.5 cm), each centred on the predefined exposure area for larvae as described in S2 Fig. (B) Illustration of the uniform deposition on a petri dish after a single pump stroke. Thiovit Jet® at 100 % concentration is shown as an example.
