# Supplementary material for: Exposure route mediates toxicological effects of sulphur and fluxapyroxad fungicides in a non-target butterfly
Source: PLoS One. 2026 Jul 9;21(7):e0353528. doi: 10.1371/journal.pone.0353528 (PMC13349104; doi:10.1371/journal.pone.0353528)
Supplement: S1 Text — (DOCX) [file pone.0353528.s015.docx]

**S1 Text. Calibration of the hand sprayer for oral exposure.**

To ensure that exactly 100 ml per 1 m² were applied uniformly, the hand sprayer (Gloria 3) was calibrated prior to the experiments. Preliminary trials were conducted by spraying solutions into a measuring cup to determine the optimal fill volume that allowed for uniform deposition of 100 ml, avoiding interruption or uneven application at the end of spraying while minimizing excess solution use. The time required to deliver 100 ml was independently measured by two experimenters. Filling 150 ml into the hand sprayer and pressurizing the bottles with 50 pumps reliably delivered 100 ml in 12 s. To verify the consistency of the sprayer under experimental conditions, petri dishes with filter paper were placed into the spraying area, and cabbage leaves were positioned alongside to visually monitor uniform fungicide coverage. Filter papers were weighed before spraying, and again after 24 h and 48 h of drying under a fume hood to allow evaporation of the liquid carrier so that only the applied substance remained. Results (S2 Table) indicate that the hand sprayer delivered consistent amounts and that the spraying was applied evenly. Note that spraying was performed only by MY throughout all experiments to ensure consistency and minimize biases due to nozzle handling, sprayer positioning, or application technique.
