# Supplementary material for: Exposure route mediates toxicological effects of sulphur and fluxapyroxad fungicides in a non-target butterfly
Source: PLoS One. 2026 Jul 9;21(7):e0353528. doi: 10.1371/journal.pone.0353528 (PMC13349104; doi:10.1371/journal.pone.0353528)
Supplement: S2 Text — (DOCX) [file pone.0353528.s016.docx]

**S2 Text. Calibration of the hand sprayer and calculation of applied fungicide amounts for contact exposure.**

As for oral exposure (see S1 Text), calibration was confirmed and the applied doses quantified also for contact exposure. To ensure consistent application without irregular discharge or interruption, each sprayer was filled with 200 ml of the respective solution and pressurized with 50 pumps, allowing at least 10 consecutive spray applications (defined as a single pump stroke directed at the larva). Between individual applications, the sprayer was briefly flushed by continuous spraying to prevent air accumulation in the hose and maintain a stable and consistent spray output. To evaluate the applied fungicide dose for each fungicide and concentration, a petri dish was positioned on the predefined exposure area (S4 Fig; 10 cm x 12.5 cm). A single pump stroke was applied to the area. Prior to testing, the setup was evaluated using water to ensure uniform coverage of the predefined exposure area (example for Thiovit Jet® at 100 % concentration is given in S4B Fig). The actual amount of formulated product **(mg)** deposited per spray application per area was subsequently determined from the weight increase of the petri dish. The measured deposition was then **scaled up to the total exposure area** to estimate the applied dose per area (applied amount per dish x (dish area / exposure area); S4 Table). This procedure allowed for an accurate comparison of fungicide concentrations under controlled conditions. Variation in delivered doses can occur due to factors such as increased solution viscosity at high concentrations, emphasizing the importance of quantifying the applied amount for reliable interpretation of contact exposure results. As above (S1 Text), spraying was performed exclusively by MY.
