# Supplementary material for: Exposure route mediates toxicological effects of sulphur and fluxapyroxad fungicides in a non-target butterfly
Source: PLoS One. 2026 Jul 9;21(7):e0353528. doi: 10.1371/journal.pone.0353528 (PMC13349104; doi:10.1371/journal.pone.0353528)
Supplement: S1 Data — (DOCX) [file pone.0353528.s017.docx]

**S1 Data. Dataset on mortality and sublethal effects of sulphur-based and fluxapyroxad fungicides in *Pieris rapae***

This dataset was generated from controlled laboratory experiments in which *Pieris rapae* larvae were exposed to sulphur-based and fluxapyroxad fungicides via both oral and contact exposure. For the oral exposure, realistic field rates of fungicides were used, whereas for the contact exposure, fungicide concentrations included field realistic as well as concentrations up to 10 times the maximum recommended field rate. Survival and sublethal endpoints, including developmental time, larval growth rate, pupal mass, morphological traits (thorax and abdomen mass, thorax–abdomen ratio, forewing length), and relative fat content, were recorded to assess the effects of fungicide exposure on a non-target lepidopteran species.

**Description:** The dataset consists of two tables: (1) Oral exposure and (2) Contact exposure. For the oral exposure experiment, survival as well as sublethal traits were assessed. For the contact exposure only survival was assessed.

**Variables:**

**Table 1. Oral exposure experiment:**

- **M_ID:** Mother ID = identifier of the mother individual
- **Treatment** = control and fungicide treatment (C = control group, T = Thiovit Jet® (sulphur-based), S = Stulln® (sulphur-based), Se = Sercadis® (active ingredient: fluxapyroxad)
- **L_ID:** Larval ID = individual larval identifier
- **Mortality** = survival status (assessed until adult eclosion; 1 = dead, 0 = alive)
- **Sex** = sex of eclosed butterflies (F = female, M = male)
- **LD_time:** Larval development time = time from egg hatch until pupation given in days
- **PD_time:** Pupal development time = time from pupation unitl adult eclosion given in days
- **Pupal_Mass** = pupal mass given in mg
- **Tho_Mass:** Thorax mass = frozen thorax mass given in mg
- **Abd_Mass:** Abdomen mass = frozen abdomen mass given in mg
- **TA_ratio:** Thorax abdomen ratio = ratio of thorax to abdomen mass (frozen)
- **Wing_Length** = length of the left forewing given in mm
- **Fat:** Relative_Fat_Content = realtive fat content (fat content / abdomen mass × 100)

**NOTE:** Larval growth rate was calculated in R (version 4.3.1 [1]) as the natural logarithm of mass gain per day (ln pupal mass / larval development time) and is not included as a separate variable in the dataset.

**Table 2. Contact exposure experiment:**

- **M_ID:** Mother ID = identifier of the mother individual
- **Treatment** = fungicide treatment (T = Thiovit Jet® (sulphur-based), S = Stulln® (sulphur-based), Se = Sercadis® (active ingredient: fluxapyroxad)
- **Concentration** = fungicide concentration; 0% represents the control treatment (no active ingredient applied).
- **L_ID:** Larval ID = individual larval identifier
- **Mortality** = survival status (assessed until adult eclosion; 1 = dead, 0 = alive)

**Table 1. Oral exposure experiment**

| **M_ID** | **Treatment** | **L_ID** | **Mortality** | **Sex** | **LD_time**  **(days)** | **PD_time**  **(days)** | **Pupal_Mass**  **(mg)** | **Tho_Mass**  **(mg)** | **Abd_Mass**  **(mg)** | **TA_ratio** | **Wing length (mm)** | **Fat**  **(%)** |
| --- | --- | --- | --- | --- | --- | --- | --- | --- | --- | --- | --- | --- |
| J7 | C | 11 | 0 | F | 17 | 8 | 120.40 | 7.80 | 18.30 | 0.43 | 21.44 | 43.75 |
| J6 | C | 4 | 0 | F | 15 | 8 |  | 6.50 | 15.60 | 0.42 | 26.63 | 43.75 |
| J4 | C | 14 | 0 | M | 18 | 8 | 124.30 | 6.00 |  |  | 19.56 |  |
| J4 | C | 15 | 0 | F | 15 | 9 | 133.70 | 6.20 | 19.50 | 0.32 | 21.27 | 47.62 |
| J6 | C | 78 | 0 | F | 12 | 7 | 115.40 | 4.20 |  |  | 18.77 |  |
| J8 | C | 41 | 1 |  | 14 | 7 | 99.20 |  |  |  |  |  |
| J6 | C | 26 | 0 | M | 21 | 6 | 97.70 | 3.60 | 16.40 | 0.22 | 16.00 | 40.86 |
| J8 | C | 110 | 0 |  | 15 | 8 | 140.00 | 5.00 | 14.10 | 0.35 |  | 43.01 |
| J7 | C | 62 | 0 | M | 17 | 7 | 132.60 | 8.30 | 14.10 | 0.59 | 28.49 | 50.00 |
| J6 | C | 84 | 1 |  |  |  |  |  |  |  |  |  |
| J6 | C | 79 | 0 | F | 11 | 8 | 111.40 | 4.40 | 11.70 | 0.38 | 20.46 | 36.90 |
| J6 | C | 81 | 0 | F | 14 | 7 | 113.60 | 5.10 | 15.00 | 0.34 | 21.70 | 50.00 |
| J7 | C | 63 | 0 | F | 18 | 8 | 125.80 | 9.80 | 13.00 | 0.75 | 27.01 | 37.31 |
| J4 | C | 134 | 1 |  |  |  |  |  |  |  |  |  |
| J6 | C | 310 | 0 | F | 17 | 9 | 81.30 | 3.20 | 12.50 | 0.26 | 20.33 | 49.32 |
| J8 | C | 101 | 0 | M | 16 | 8 | 138.60 | 5.80 | 23.80 | 0.24 | 22.77 | 40.52 |
| J5 | C | 33 | 0 | F | 14 | 9 | 135.20 | 2.90 | 15.70 | 0.18 | 26.06 | 42.86 |
| J8 | C | 104 | 0 | F | 14 | 9 | 103.70 | 4.70 | 11.40 | 0.41 | 26.41 | 51.61 |
| J4 | C | 135 | 0 | M | 17 | 7 | 144.50 | 6.70 | 19.90 | 0.34 | 22.78 | 61.90 |
| J6 | C | 83 | 0 | F | 13 | 7 | 109.00 | 4.10 | 12.10 | 0.34 | 19.38 | 53.70 |
| J8 | C | 102 | 0 | M | 14 | 8 | 135.40 | 9.90 | 17.30 | 0.57 | 22.87 | 44.59 |
| J4 | C | 133 | 0 | M | 17 | 7 | 132.30 | 5.60 | 15.10 | 0.37 | 18.51 | 53.95 |
| J11 | C | 91 | 0 | M | 15 | 8 | 136.30 | 5.20 | 20.90 | 0.25 | 21.50 | 49.09 |
| J7 | C | 59 | 0 | F | 16 | 8 | 128.10 | 7.70 | 28.80 | 0.27 | 28.04 | 38.40 |
| J6 | C | 71 | 0 | F | 11 | 8 | 126.20 | 5.40 | 17.40 | 0.31 | 21.49 | 47.83 |
| J8 | C | 96 | 0 | M | 17 | 7 | 140.40 | 9.80 |  |  | 27.28 |  |
| J4 | C | 128 | 1 |  |  |  |  |  |  |  |  |  |
| J6 | C | 74 | 0 | F | 14 | 8 | 113.80 | 4.10 | 15.30 | 0.27 | 20.87 | 45.33 |
| J5 | C | 38 | 1 |  |  |  |  |  |  |  |  |  |
| J9 | C | 23 | 0 | F | 16 | 8 | 112.80 | 4.80 | 21.80 | 0.22 | 20.93 | 42.31 |
| J15 | C | 165 | 1 |  |  |  |  |  |  |  |  |  |
| J15 | C | 170 | 1 |  |  |  |  |  |  |  |  |  |
| J15 | C | 194 | 1 |  |  |  |  |  |  |  |  |  |
| J15 | C | 175 | 1 |  |  |  |  |  |  |  |  |  |
| J13 | C | 156 | 0 | M | 15 | 8 | 120.40 | 3.60 | 13.50 | 0.27 | 27.68 | 44.83 |
| J15 | C | 167 | 1 |  |  |  |  |  |  |  |  |  |
| J13 | C | 154 | 1 |  |  |  |  |  |  |  |  |  |
| J15 | C | 210 | 1 |  |  |  |  |  |  |  |  |  |
| J8 | C | 180 | 1 |  |  |  |  |  |  |  |  |  |
| J9 | C | 146 | 0 | M | 16 | 7 | 119.00 | 4.80 | 13.60 | 0.35 | 21.13 | 51.43 |
| J13 | C | 153 | 0 | M | 13 | 7 | 113.20 | 5.10 | 15.40 | 0.33 | 21.22 | 46.43 |
| J8 | C | 181 | 0 | F | 14 | 8 | 129.70 | 4.50 | 23.70 | 0.19 | 27.71 | 29.33 |
| J13 | C | 155 | 0 | F | 14 | 8 | 93.50 | 2.70 | 12.20 | 0.22 | 24.31 | 38.18 |
| J15 | C | 211 | 0 | M | 13 | 8 | 138.70 | 6.10 | 17.90 | 0.34 | 28.62 | 54.17 |
| J2 | C | 236 | 0 | M | 16 | 7 | 141.20 | 5.60 | 24.70 | 0.23 | 21.97 | 60.71 |
| J15 | C | 241 | 1 |  |  |  |  |  |  |  |  |  |
| J15 | C | 242 | 0 | F | 18 | 7 | 107.60 | 4.40 | 20.80 | 0.21 | 25.43 | 50.54 |
| J8 | C | 306 | 1 |  |  |  |  |  |  |  |  |  |
| J13 | C | 237 | 1 |  |  |  |  |  |  |  |  |  |
| J5 | C | 300 | 1 |  |  |  |  |  |  |  |  |  |
| J8 | C | 271 | 0 | M | 12 | 8 | 131.20 | 6.40 | 14.00 | 0.46 | 22.40 | 46.15 |
| J8 | C | 270 | 1 |  |  |  |  |  |  |  |  |  |
| J8 | C | 272 | 1 |  | 16 |  | 126.30 |  |  |  |  |  |
| J6 | C | 590 | 0 | M | 12 | 9 | 114.80 | 4.80 | 16.70 | 0.29 | 20.69 | 50.00 |
| J5 | C | 265 | 0 | M | 17 | 7 | 106.50 | 2.80 | 22.60 | 0.12 | 25.34 | 51.04 |
| J15 | C | 267 | 1 |  |  |  |  |  |  |  |  |  |
| J9 | C | 526 | 1 |  |  |  |  |  |  |  |  |  |
| J5 | C | 451 | 1 |  |  |  |  |  |  |  |  |  |
| J7 | C | 494 | 0 | M | 15 | 7 | 146.90 | 10.90 | 11.40 | 0.96 | 29.82 | 54.10 |
| J14 | C | 419 | 0 | M | 15 | 7 | 136.40 | 5.50 | 17.90 | 0.31 | 28.88 | 53.95 |
| J7 | C | 483 | 1 |  |  |  |  |  |  |  |  |  |
| J5 | C | 443 | 1 |  |  |  |  |  |  |  |  |  |
| J9 | C | 527 | 1 |  |  |  |  |  |  |  |  |  |
| J1 | C | 411 | 0 | M | 11 | 9 | 127.30 | 6.10 | 19.10 | 0.32 | 27.47 | 36.67 |
| J5 | C | 456 | 0 | F | 13 | 7 | 111.40 | 4.90 | 16.40 | 0.30 | 20.70 | 53.57 |
| J9 | C | 544 | 0 | M | 15 | 7 | 111.40 | 4.00 | 23.10 | 0.17 | 28.78 | 51.58 |
| J9 | C | 522 | 1 |  |  |  |  |  |  |  |  |  |
| J6 | C | 511 | 1 |  |  |  |  |  |  |  |  |  |
| J5 | C | 464 | 0 | M | 15 | 7 | 111.40 | 2.60 | 13.30 | 0.20 | 24.11 | 52.63 |
| J5 | C | 409 | 0 | F | 11 | 8 | 122.70 | 5.00 | 22.30 | 0.22 | 21.95 | 50.91 |
| J6 | C | 144 | 0 | M | 20 | 7 | 111.40 | 3.30 | 26.90 | 0.12 | 18.37 | 46.39 |
| J1 | C | 476 | 0 | M | 16 | 7 | 116.70 | 4.70 | 21.70 | 0.22 | 21.42 | 39.78 |
| J11 | C | 431 | 0 | M | 16 | 7 | 137.90 | 5.50 | 9.60 | 0.57 | 21.13 | 48.15 |
| J9 | C | 537 | 1 |  |  |  |  |  |  |  |  |  |
| J5 | C | 452 | 0 | F | 14 | 7 | 124.10 | 7.90 | 19.70 | 0.40 | 25.79 | 52.38 |
| J14 | C | 572 | 1 |  | 15 |  | 120.20 |  |  |  |  |  |
| J7 | C | 564 | 1 |  |  |  |  |  |  |  |  |  |
| J3 | C | 553 | 1 |  |  |  |  |  |  |  |  |  |
| J8 | C | 587 | 0 | M | 16 | 7 | 132.40 | 6.00 | 21.50 | 0.28 | 27.28 | 47.27 |
| J1 | C | 559 | 1 |  | 14 |  | 116.80 |  |  |  |  |  |
| J3 | C | 596 | 0 | M | 15 | 7 | 133.50 | 5.70 | 15.50 | 0.37 | 21.77 | 48.78 |
| J14 | C | 578 | 1 |  |  |  |  |  |  |  |  |  |
| J14 | C | 576 | 1 |  |  |  |  |  |  |  |  |  |
| J14 | C | 649 | 1 |  | 14 |  | 139.80 |  |  |  |  |  |
| J22 | C | 684 | 1 |  |  |  |  |  |  |  |  |  |
| J22 | C | 660 | 1 |  |  |  |  |  |  |  |  |  |
| J22 | C | 695 | 1 |  |  |  |  |  |  |  |  |  |
| J22 | C | 674 | 1 | F | 14 | 7 | 112.20 | 2.80 | 13.10 | 0.21 | 24.36 | 47.30 |
| J14 | C | 648 | 1 |  |  |  |  |  |  |  |  |  |
| J19 | C | 631 | 1 |  |  |  |  |  |  |  |  |  |
| J22 | C | 673 | 1 |  |  |  |  |  |  |  |  |  |
| J22 | C | 653 | 1 |  | 15 |  | 142.30 |  |  |  |  |  |
| J22 | C | 633 | 0 | F | 14 | 7 | 119.00 | 4.30 | 24.00 | 0.18 | 28.01 | 41.67 |
| J19 | C | 700 | 1 |  |  |  |  |  |  |  |  |  |
| J22 | C | 687 | 1 |  |  |  |  |  |  |  |  |  |
| J19 | C | 625 | 0 | M | 16 | 7 | 125.50 | 3.70 |  |  | 20.20 |  |
| J22 | C | 672 | 1 |  |  |  |  |  |  |  |  |  |
| J14 | C | 681 | 1 |  |  |  |  |  |  |  |  |  |
| J14 | C | 679 | 1 |  |  |  |  |  |  |  |  |  |
| J7 | T | 10 | 1 |  |  |  |  |  |  |  |  |  |
| J6 | T | 5 | 0 | F | 14 | 10 | 102.50 | 4.10 | 18.70 | 0.22 | 24.03 | 58.62 |
| J8 | T | 19 | 0 | F | 14 | 9 | 129.00 | 5.10 | 23.70 | 0.22 | 27.44 | 42.73 |
| J5 | T | 3 | 0 | F | 15 | 10 | 99.10 | 4.80 | 19.60 | 0.24 | 18.09 | 47.52 |
| J8 | T | 312 | 0 | M | 13 | 9 | 136.60 | 7.60 | 15.20 | 0.50 | 25.04 | 45.59 |
| J4 | T | 129 | 0 | M | 17 | 9 | 101.90 | 2.50 |  |  | 16.51 |  |
| J8 | T | 103 | 0 | M | 18 | 9 | 140.60 | 5.90 | 20.00 | 0.30 | 21.72 | 47.17 |
| J7 | T | 61 | 0 | F | 16 | 8 | 128.50 | 5.30 | 18.50 | 0.29 | 21.73 | 41.49 |
| J6 | T | 75 | 0 | M | 11 | 9 | 112.10 | 3.40 | 15.60 | 0.22 | 24.36 | 39.08 |
| J6 | T | 80 | 0 | M | 12 | 8 | 112.90 | 3.90 | 14.90 | 0.26 | 24.05 | 40.74 |
| J7 | T | 60 | 0 | F | 16 | 10 | 131.70 | 6.10 | 25.00 | 0.24 | 28.49 | 40.18 |
| J8 | T | 111 | 0 | F | 15 | 10 | 128.60 | 5.30 | 21.60 | 0.25 | 28.40 | 40.43 |
| J10 | T | 121 | 0 | F | 13 | 8 | 132.40 | 5.50 | 15.60 | 0.35 | 27.61 | 55.56 |
| J8 | T | 44 | 0 | M | 15 | 9 | 139.70 | 4.60 | 23.30 | 0.20 | 29.72 | 39.82 |
| J5 | T | 45 | 0 | F | 14 | 10 | 113.20 | 5.00 | 20.40 | 0.25 | 20.42 | 48.51 |
| J5 | T | 34 | 0 | F | 14 | 10 | 119.80 | 3.80 | 22.90 | 0.17 | 26.70 | 47.22 |
| J6 | T | 90 | 0 | M | 16 | 9 | 119.80 | 3.80 | 15.80 | 0.24 | 21.27 | 43.04 |
| J7 | T | 64 | 0 | F | 18 | 9 | 140.50 | 3.90 | 16.70 | 0.23 | 23.14 | 48.84 |
| J8 | T | 42 | 0 | M | 15 | 9 | 138.90 | 6.20 | 16.80 | 0.37 | 27.38 | 42.31 |
| J8 | T | 100 | 0 |  | 14 | 14 | 125.90 |  |  |  |  |  |
| J8 | T | 112 | 0 | F | 17 | 8 | 133.20 | 4.70 | 13.00 | 0.36 | 22.08 | 38.20 |
| J11 | T | 117 | 0 | F | 14 | 9 | 131.30 | 8.20 | 18.50 | 0.44 | 28.07 | 59.49 |
| J6 | T | 48 | 0 | F | 14 | 9 | 109.40 | 5.10 | 16.10 | 0.32 | 26.07 | 49.43 |
| J9 | T | 22 | 1 |  |  |  |  |  |  |  |  |  |
| J8 | T | 107 | 0 | F | 16 | 9 | 124.50 | 5.00 | 15.10 | 0.33 | 27.29 | 52.87 |
| J8 | T | 113 | 0 | F | 17 | 10 | 150.70 | 5.00 | 26.50 | 0.19 | 22.62 | 30.23 |
| J8 | T | 106 | 0 | F | 17 | 8 | 126.70 | 4.60 | 15.10 | 0.30 | 21.90 | 48.05 |
| J5 | T | 32 | 0 | M | 14 | 9 | 140.40 | 9.80 | 20.50 | 0.48 | 29.38 | 48.24 |
| J8 | T | 108 | 0 | F | 16 | 10 | 116.00 | 5.40 | 17.90 | 0.30 | 28.12 | 43.02 |
| J11 | T | 190 | 1 |  |  |  |  |  |  |  |  |  |
| J15 | T | 143 | 0 | M | 16 | 11 | 109.00 | 5.30 | 17.70 | 0.30 | 23.34 | 50.00 |
| J3 | T | 226 | 0 | F | 16 | 9 | 100.00 | 4.50 | 13.60 | 0.33 | 20.03 | 50.70 |
| J3 | T | 227 | 1 |  |  |  |  |  |  |  |  |  |
| J5 | T | 216 | 0 | M | 16 | 8 | 132.10 | 6.30 | 14.60 | 0.43 | 28.21 | 51.25 |
| J15 | T | 171 | 0 | M | 14 | 10 | 146.00 | 4.60 | 22.40 | 0.21 | 22.47 | 48.11 |
| J5 | T | 218 | 0 | F | 15 | 9 | 109.90 | 4.30 | 17.50 | 0.25 | 19.87 | 52.69 |
| J6 | T | 145 | 1 |  |  |  |  |  |  |  |  |  |
| J11 | T | 187 | 1 |  |  |  |  |  |  |  |  |  |
| J15 | T | 195 | 0 | M | 13 | 9 | 116.00 | 8.50 |  |  | 26.12 |  |
| J3 | T | 224 | 0 | M | 16 | 9 | 104.00 | 5.40 | 17.70 | 0.31 | 23.21 | 40.74 |
| J5 | T | 147 | 1 |  |  |  |  |  |  |  |  |  |
| J5 | T | 219 | 0 | F | 17 | 9 | 126.00 | 5.30 | 18.50 | 0.29 | 21.75 | 40.63 |
| J5 | T | 220 | 1 |  | 16 |  | 113.60 |  |  |  |  |  |
| J6 | T | 278 | 0 | F | 15 | 9 | 97.30 | 4.10 | 14.40 | 0.28 | 24.14 | 42.25 |
| J1 | T | 296 | 0 | M | 16 | 8 | 114.20 | 4.60 | 16.10 | 0.29 | 26.56 | 33.78 |
| J13 | T | 710 | 0 | F | 17 | 8 | 118.50 | 4.20 | 15.70 | 0.27 | 22.13 | 44.12 |
| J5 | T | 263 | 0 | M | 14 | 9 | 113.30 | 4.30 | 14.70 | 0.29 | 20.44 | 40.28 |
| J5 | T | 264 | 1 |  |  |  |  |  |  |  |  |  |
| J13 | T | 305 | 1 |  | 13 |  | 120.40 |  |  |  |  |  |
| J11 | T | 245 | 0 | M | 15 | 8 | 130.50 | 5.20 | 18.90 | 0.28 | 22.75 | 47.87 |
| J15 | T | 269 | 1 |  |  |  |  |  |  |  |  |  |
| J6 | T | 282 | 0 | M | 15 | 9 | 119.50 | 5.00 | 18.40 | 0.27 | 26.62 | 47.25 |
| J6 | T | 284 | 0 | F | 16 | 9 | 105.40 | 3.60 | 20.80 | 0.17 | 19.33 | 41.05 |
| J6 | T | 277 | 0 | F | 12 | 10 | 114.50 | 4.20 | 23.00 | 0.18 | 20.34 | 35.24 |
| J5 | T | 303 | 1 |  |  |  |  |  |  |  |  |  |
| J13 | T | 305 | 0 | W | 22 | 13 | 9.00 |  |  |  | 0.00 |  |
| J15 | T | 591 | 1 |  |  |  |  |  |  |  |  |  |
| J7 | T | 410 | 0 | F | 16 | 8 | 140.50 | 6.40 | 20.20 | 0.32 | 29.43 | 44.66 |
| J9 | T | 525 | 1 |  |  |  |  |  |  |  |  |  |
| J6 | T | 507 | 1 |  |  |  |  |  |  |  |  |  |
| J1 | T | 475 | 0 | F | 14 | 9 | 120.90 | 5.00 | 15.70 | 0.32 | 25.78 | 28.17 |
| J9 | T | 533 | 1 |  |  |  |  |  |  |  |  |  |
| J6 | T | 512 | 1 |  |  |  |  |  |  |  |  |  |
| J9 | T | 545 | 1 |  |  |  |  |  |  |  |  |  |
| J1 | T | 423 | 0 | M | 15 | 8 | 142.00 | 4.90 | 19.70 | 0.25 | 22.37 | 42.27 |
| J5 | T | 439 | 0 | M | 15 | 8 | 118.70 | 5.00 |  |  |  |  |
| J14 | T | 416 | 1 |  |  |  |  |  |  |  |  |  |
| J10 | T | 422 | 0 | M | 12 | 9 | 138.00 | 5.10 |  |  | 28.89 |  |
| J6 | T | 505 | 1 |  |  |  |  |  |  |  |  |  |
| J9 | T | 529 | 1 |  |  |  |  |  |  |  |  |  |
| J9 | T | 513 | 0 | M | 18 | 8 | 105.10 | 4.10 | 15.60 | 0.26 | 18.13 | 49.38 |
| J11 | T | 430 | 1 |  | 13 |  | 151.40 |  |  |  |  |  |
| J7 | T | 495 | 1 |  |  |  |  |  |  |  |  |  |
| J22 | T | 745 | 0 | M | 18 | 9 | 116.80 | 3.30 | 19.60 | 0.17 | 27.10 | 37.50 |
| J5 | T | 453 | 1 |  |  |  |  |  |  |  |  |  |
| J9 | T | 523 | 1 |  |  |  |  |  |  |  |  |  |
| J9 | T | 542 | 0 | F | 17 | 8 | 104.60 | 3.60 | 13.10 | 0.27 | 26.28 | 24.14 |
| J7 | T | 500 | 0 | M | 16 | 10 | 119.80 | 4.50 | 13.50 | 0.33 | 25.77 | 44.87 |
| J11 | T | 707 | 0 | M | 17 | 8 | 142.50 | 5.60 | 20.20 | 0.28 | 28.49 | 45.36 |
| J11 | T | 586 | 0 | M | 17 | 7 | 146.10 | 5.20 | 21.90 | 0.24 | 20.90 | 50.54 |
| J14 | T | 573 | 0 | M | 15 | 9 | 132.10 | 4.10 | 17.10 | 0.24 | 21.90 | 47.31 |
| J14 | T | 557 | 0 | F | 16 | 8 | 126.90 | 5.50 | 26.90 | 0.20 | 21.58 | 41.43 |
| J7 | T | 567 | 0 | F | 15 | 9 | 129.20 | 5.50 | 16.70 | 0.33 | 29.08 | 58.06 |
| J8 | T | 588 | 1 |  |  |  |  |  |  |  |  |  |
| J14 | T | 580 | 1 |  | 12 |  | 140.00 |  |  |  |  |  |
| J14 | T | 577 | 0 | M | 13 | 8 | 142.80 | 4.80 | 26.90 | 0.18 | 22.15 | 38.33 |
| J14 | T | 621 | 1 |  |  |  |  |  |  |  |  |  |
| J22 | T | 646 | 1 |  |  |  |  |  |  |  |  |  |
| J22 | T | 667 | 0 | M | 15 | 8 | 161.20 | 7.10 | 19.70 | 0.36 | 29.57 | 45.92 |
| J22 | T | 657 | 1 |  |  |  |  |  |  |  |  |  |
| J22 | T | 693 | 0 | M | 16 | 9 | 130.80 | 4.80 | 19.00 | 0.25 | 22.16 | 45.28 |
| J22 | T | 694 | 1 |  |  |  |  |  |  |  |  |  |
| J22 | T | 701 | 1 |  |  |  |  |  |  |  |  |  |
| J22 | T | 676 | 1 |  |  |  |  |  |  |  |  |  |
| J19 | T | 628 | 1 |  |  |  |  |  |  |  |  |  |
| J22 | T | 666 | 1 |  |  |  |  |  |  |  |  |  |
| J22 | T | 690 | 0 | F | 14 | 8 | 174.90 | 6.20 | 29.70 | 0.21 | 31.92 | 42.66 |
| J8 | S | 7 | 1 |  |  |  |  |  |  |  |  |  |
| J4 | S | 17 | 0 | M | 15 | 10 | 128.90 | 5.70 | 20.50 | 0.28 | 19.47 | 45.28 |
| J7 | S | 12 | 0 | F | 17 | 10 | 157.00 | 6.40 | 22.60 | 0.28 | 29.71 | 36.94 |
| J4 | S | 132 | 0 | F | 14 | 9 | 116.40 | 5.00 | 14.40 | 0.35 | 21.45 | 42.53 |
| J5 | S | 35 | 0 | F | 16 | 10 | 100.50 | 3.00 | 10.30 | 0.29 | 18.50 | 39.29 |
| J6 | S | 53 | 0 | F | 14 | 9 | 104.60 | 4.30 | 21.60 | 0.20 | 26.06 | 50.00 |
| J6 | S | 25 | 0 | F | 14 | 9 | 121.90 | 4.90 | 21.10 | 0.23 | 21.79 | 42.57 |
| J11 | S | 551 | 0 | F | 12 | 8 | 139.40 | 4.90 |  |  |  |  |
| J6 | S | 72 | 0 | M | 14 | 9 | 129.20 | 5.30 | 23.40 | 0.23 | 21.57 | 46.85 |
| J6 | S | 311 | 0 | F | 16 | 10 | 119.80 | 4.20 | 24.70 | 0.17 | 25.81 | 50.83 |
| J8 | S | 97 | 0 | M | 15 | 9 | 134.30 | 5.90 | 20.60 | 0.29 | 22.37 | 50.00 |
| J6 | S | 89 | 0 | F | 14 | 9 | 117.80 | 4.20 | 18.60 | 0.23 | 20.23 | 49.48 |
| J5 | S | 31 | 1 |  |  |  |  |  |  |  |  |  |
| J5 | S | 123 | 1 |  |  |  |  |  |  |  |  |  |
| J8 | S | 94 | 0 | F | 14 | 8 | 97.20 | 3.10 | 17.10 | 0.18 | 23.37 | 48.84 |
| J5 | S | 46 | 1 |  |  |  |  |  |  |  |  |  |
| J8 | S | 109 | 0 | F | 18 | 9 | 103.50 | 4.50 | 15.00 | 0.30 | 25.57 | 43.94 |
| J11 | S | 118 | 0 | M | 14 | 9 | 145.30 | 5.00 | 18.20 | 0.27 | 22.67 | 53.33 |
| J5 | S | 124 | 0 | F | 17 | 9 | 118.90 | 5.00 |  |  | 20.51 |  |
| J6 | S | 51 | 0 | F | 13 | 9 | 118.70 | 4.50 | 21.50 | 0.21 | 21.11 | 52.43 |
| J4 | S | 131 | 0 | F | 15 | 9 | 101.80 | 4.70 |  |  | 19.53 |  |
| J3 | S | 316 | 1 |  |  |  |  |  |  |  |  |  |
| J8 | S | 140 | 0 | F | 15 | 10 | 156.90 | 5.10 | 27.40 | 0.19 | 23.25 | 44.54 |
| J11 | S | 93 | 0 | F | 14 | 9 | 134.60 | 4.90 | 22.10 | 0.22 | 29.21 | 55.05 |
| J5 | S | 36 | 1 |  | 11 |  | 120.70 |  |  |  |  |  |
| J7 | S | 308 | 0 | F | 14 | 9 | 140.70 | 6.80 | 18.60 | 0.37 | 22.03 | 36.67 |
| J5 | S | 148 | 1 |  |  |  |  |  |  |  |  |  |
| J11 | S | 188 | 1 |  |  |  |  |  |  |  |  |  |
| J5 | S | 149 | 1 |  |  |  |  |  |  |  |  |  |
| J11 | S | 189 | 0 | F | 13 | 9 | 102.80 | 4.00 | 14.30 | 0.28 | 19.46 | 39.44 |
| J3 | S | 230 | 1 |  | 17 |  | 107.90 |  |  |  |  |  |
| J15 | S | 172 | 0 | F | 14 | 9 | 128.00 | 4.20 | 21.00 | 0.20 | 21.13 | 48.08 |
| J3 | S | 228 | 1 |  |  |  |  |  |  |  |  |  |
| J15 | S | 173 | 1 |  |  |  |  |  |  |  |  |  |
| J3 | S | 231 | 0 | F | 17 | 8 | 102.10 | 3.60 | 17.00 | 0.21 | 20.60 | 48.33 |
| J11 | S | 192 | 1 |  |  |  |  |  |  |  |  |  |
| J15 | S | 202 | 0 | M | 16 | 8 | 122.90 | 3.70 | 14.30 | 0.26 | 19.07 | 44.62 |
| J15 | S | 200 | 1 |  |  |  |  |  |  |  |  |  |
| J15 | S | 201 | 1 |  |  |  |  |  |  |  |  |  |
| J5 | S | 259 | 0 | F | 14 | 9 | 130.00 | 6.10 | 14.90 | 0.41 | 28.82 | 48.81 |
| J5 | S | 260 | 0 | F | 15 | 9 | 126.50 | 5.80 | 20.10 | 0.29 | 20.82 | 48.08 |
| J5 | S | 261 | 1 |  |  |  |  |  |  |  |  |  |
| J5 | S | 262 | 0 | M | 15 | 8 | 117.00 | 4.20 | 13.50 | 0.31 | 20.57 | 50.00 |
| J5 | S | 258 | 0 | M | 15 | 9 | 105.00 | 4.50 | 12.00 | 0.38 | 24.59 | 49.15 |
| J11 | S | 254 | 1 |  |  |  |  |  |  |  |  |  |
| J6 | S | 279 | 0 | M | 13 | 9 | 114.50 | 4.30 | 12.40 | 0.35 | 19.82 | 53.25 |
| J1 | S | 299 | 0 | F | 13 | 9 | 123.10 | 5.00 | 16.30 | 0.31 | 20.99 | 42.68 |
| J1 | S | 298 | 0 | F | 12 | 9 | 120.40 | 4.20 | 21.20 | 0.20 | 27.32 | 36.96 |
| J11 | S | 244 | 0 | F | 14 | 9 | 131.10 | 5.50 | 16.40 | 0.34 | 27.56 | 40.00 |
| J11 | S | 255 | 1 |  |  |  |  |  |  |  |  |  |
| J11 | S | 253 | 0 | M | 15 | 9 | 134.20 | 4.20 | 17.30 | 0.24 | 21.43 | 52.56 |
| J11 | S | 252 | 0 | M | 15 | 9 | 137.80 | 5.30 |  |  | 20.92 |  |
| J11 | S | 251 | 1 |  |  |  |  |  |  |  |  |  |
| J9 | S | 546 | 0 | M | 18 | 8 | 116.30 | 4.40 | 20.20 | 0.22 |  | 46.84 |
| J7 | S | 484 | 1 |  |  |  |  |  |  |  |  |  |
| J5 | S | 455 | 0 | F | 13 | 10 | 137.50 | 6.80 | 17.30 | 0.39 | 29.16 | 44.62 |
| J6 | S | 506 | 1 |  |  |  |  |  |  |  |  |  |
| J9 | S | 528 | 1 |  |  |  |  |  |  |  |  |  |
| J9 | S | 514 | 1 |  |  |  |  |  |  |  |  |  |
| J1 | S | 477 | 1 |  |  |  |  |  |  |  |  |  |
| J9 | S | 538 | 0 | F | 16 | 9 | 139.60 | 5.20 | 20.20 | 0.26 | 21.57 | 43.02 |
| J5 | S | 465 | 1 |  | 14 |  | 132.90 |  |  |  |  |  |
| J14 | S | 413 | 1 |  |  |  |  |  |  |  |  |  |
| J9 | S | 531 | 0 | M | 16 | 8 | 103.60 | 4.20 | 18.20 | 0.23 | 26.10 | 40.23 |
| J5 | S | 441 | 1 |  |  |  |  |  |  |  |  |  |
| J1 | S | 424 | 1 |  |  | 10 |  |  |  |  |  |  |
| J14 | S | 417 | 1 |  |  |  |  |  |  |  |  |  |
| J5 | S | 442 | 1 |  |  |  |  |  |  |  |  |  |
| J14 | S | 414 | 0 | F | 13 | 8 | 114.80 | 4.00 | 20.80 | 0.19 | 21.14 | 51.96 |
| J5 | S | 438 | 0 | M | 13 |  | 115.80 | 5.60 | 12.70 | 0.44 | 24.85 | 53.23 |
| J5 | S | 472 | 0 | F | 15 | 10 | 136.10 | 4.70 | 19.10 | 0.25 | 27.58 | 42.45 |
| J5 | S | 471 | 1 |  |  |  |  |  |  |  |  |  |
| J7 | S | 496 | 0 | F | 13 | 9 | 126.70 | 6.10 | 17.10 | 0.36 | 21.19 | 43.66 |
| J5 | S | 467 | 0 | F | 14 | 9 | 108.30 | 4.50 | 21.90 | 0.21 | 26.55 | 49.54 |
| J3 | S | 556 | 1 |  |  |  |  |  |  |  |  |  |
| J7 | S | 570 | 0 | F | 20 | 9 | 121.30 | 5.10 | 16.50 | 0.31 | 26.31 | 50.00 |
| J7 | S | 565 | 1 |  |  |  |  |  |  |  |  |  |
| J9 | S | 585 | 1 |  |  |  |  |  |  |  |  |  |
| J8 | S | 552 | 1 |  |  |  |  |  |  |  |  |  |
| J14 | S | 575 | 0 | F | 13 | 8 | 114.90 | 5.10 |  |  | 20.15 |  |
| J3 | S | 597 | 1 |  |  |  |  |  |  |  |  |  |
| J3 | S | 594 | 1 |  |  |  |  |  |  |  |  |  |
| J19 | S | 626 | 1 |  |  |  |  |  |  |  |  |  |
| J14 | S | 680 | 1 |  | 15 |  | 112.40 |  |  |  |  |  |
| J22 | S | 641 | 1 |  |  |  |  |  |  |  |  |  |
| J22 | S | 652 | 1 |  |  | 10 |  |  |  |  |  |  |
| J14 | S | 651 | 1 |  |  |  |  |  |  |  |  |  |
| J19 | S | 627 | 1 |  |  |  |  |  |  |  |  |  |
| J22 | S | 661 | 1 |  |  |  |  |  |  |  |  |  |
| J22 | S | 686 | 0 | F | 14 | 8 | 116.50 | 3.60 | 20.40 | 0.18 |  | 44.23 |
| J22 | S | 636 | 1 |  |  |  |  |  |  |  |  |  |
| J22 | S | 656 | 1 |  |  |  |  |  |  |  |  |  |
| J22 | S | 668 | 1 |  |  |  |  |  |  |  |  |  |
| J22 | S | 654 | 1 |  |  |  |  |  |  |  |  |  |
| J7 | S | 663 | 1 |  |  |  |  |  |  |  |  |  |
| J22 | S | 634 | 1 |  | 16 |  | 108.70 |  |  |  |  |  |
| J22 | S | 642 | 0 | M | 15 | 8 | 163.50 | 4.60 | 30.00 | 0.15 | 23.36 | 62.93 |
| J22 | S | 645 | 1 |  | 13 |  | 159.00 |  |  |  |  |  |
| J6 | Se | 6 | 0 | F | 13 | 8 | 134.50 | 5.90 | 17.70 | 0.33 | 21.42 | 38.64 |
| J8 | Se | 8 | 1 |  | 14 |  | 121.30 |  |  |  |  |  |
| J6 | Se | 5 | 0 | M | 15 | 7 | 140.20 | 6.20 |  |  | 27.96 |  |
| J4 | Se | 16 | 0 | M | 15 | 7 | 124.30 | 4.80 | 14.80 | 0.32 | 26.02 | 48.05 |
| J8 | Se | 99 | 0 | F | 15 | 7 | 115.80 | 5.00 | 17.60 | 0.28 | 26.88 | 46.43 |
| J3 | Se | 315 | 0 | F | 16 | 7 | 107.80 | 4.20 | 14.40 | 0.29 | 20.41 | 56.58 |
| J6 | Se | 73 | 0 | F | 13 | 7 | 110.60 | 4.30 | 15.70 | 0.27 | 25.26 | 60.71 |
| J5 | Se | 30 | 0 | M | 14 | 8 | 123.20 | 5.10 | 18.80 | 0.27 | 25.80 | 51.61 |
| J3 | Se | 137 | 0 | F | 16 | 8 | 128.20 | 5.50 | 22.70 | 0.24 | 21.62 | 53.10 |
| J8 | Se | 98 | 0 | M | 14 | 7 | 142.00 | 5.80 | 22.40 | 0.26 | 28.01 | 48.91 |
| J6 | Se | 50 | 0 | M | 15 | 7 | 119.50 | 4.90 | 18.30 | 0.27 | 25.97 | 53.61 |
| J6 | Se | 49 | 1 |  |  |  |  |  |  |  |  |  |
| J5 | Se | 29 | 1 |  |  |  |  |  |  |  |  |  |
| J4 | Se | 130 | 1 |  |  |  |  |  |  |  |  |  |
| J8 | Se | 43 | 0 | M | 14 | 8 | 135.20 | 6.30 | 15.40 | 0.41 | 26.29 | 47.30 |
| J9 | Se | 20 | 0 | F | 13 | 8 | 124.10 | 4.90 | 23.10 | 0.21 | 26.52 | 45.95 |
| J8 | Se | 142 | 0 | M | 14 | 7 | 144.80 | 6.50 | 21.00 | 0.31 | 30.04 | 49.50 |
| J8 | Se | 95 | 0 | M | 15 | 7 | 120.00 | 5.10 | 17.60 | 0.29 | 22.15 | 45.33 |
| J6 | Se | 24 | 0 | F | 15 | 8 | 107.20 | 3.70 | 20.50 | 0.18 | 24.03 | 51.02 |
| J8 | Se | 141 | 0 | M | 13 | 7 | 115.60 | 4.50 | 12.60 | 0.36 | 25.90 | 37.74 |
| J5 | Se | 318 | 0 | M | 14 | 8 | 138.80 | 5.60 | 24.70 | 0.23 | 22.67 | 41.74 |
| J6 | Se | 82 | 0 | F | 14 | 7 | 120.40 | 4.70 | 17.30 | 0.27 | 26.42 | 53.09 |
| J11 | Se | 92 | 0 | M | 14 | 7 | 145.80 | 7.00 | 18.30 | 0.38 | 29.98 | 44.44 |
| J3 | Se | 138 | 0 | M | 15 | 8 | 131.60 | 5.40 | 18.10 | 0.30 | 20.82 | 48.39 |
| J5 | Se | 39 | 0 | M | 14 | 7 | 140.50 | 6.10 | 22.80 | 0.27 | 29.50 | 41.82 |
| J4 | Se | 127 | 0 | M | 14 | 8 | 119.00 | 5.00 | 12.90 | 0.39 | 24.39 | 64.47 |
| J7 | Se | 28 | 0 | F | 17 | 7 | 105.70 | 3.90 | 13.80 | 0.28 | 25.72 | 52.24 |
| J15 | Se | 166 | 0 | M | 13 | 8 | 125.40 | 4.80 | 21.30 | 0.23 | 20.41 | 43.43 |
| J15 | Se | 169 | 0 | M | 13 | 8 |  | 4.30 | 19.10 | 0.23 | 25.02 | 37.50 |
| J15 | Se | 174 | 1 |  |  |  |  |  |  |  |  |  |
| J6 | Se | 163 | 0 | F | 14 | 7 | 120.00 | 4.90 | 19.60 | 0.25 | 21.07 | 44.66 |
| J8 | Se | 179 | 0 | M | 13 | 7 | 141.90 | 6.00 | 25.30 | 0.24 | 22.59 | 41.53 |
| J15 | Se | 164 | 1 |  |  |  |  |  |  |  |  |  |
| J15 | Se | 168 | 1 |  |  |  |  |  |  |  |  |  |
| J13 | Se | 157 | 0 | M | 13 | 7 | 118.40 | 4.40 | 16.40 | 0.27 | 28.01 | 44.00 |
| J11 | Se | 191 | 1 |  |  |  |  |  |  |  |  |  |
| J7 | Se | 307 | 0 | F | 14 | 8 | 118.30 | 4.60 | 18.60 | 0.25 | 26.14 | 45.98 |
| J15 | Se | 169 | 1 |  | 13 |  | 114.20 |  |  |  |  |  |
| J1 | Se | 297 | 0 | M | 12 | 8 | 112.70 | 4.30 | 16.80 | 0.26 | 25.55 | 43.02 |
| J5 | Se | 302 | 0 | F | 14 | 7 | 106.00 | 4.40 | 17.00 | 0.26 | 25.42 | 50.55 |
| J11 | Se | 250 | 0 | F | 16 | 7 | 134.10 | 5.90 | 19.50 | 0.30 | 27.81 | 50.00 |
| J1 | Se | 292 | 0 | F | 15 | 7 | 98.30 | 3.80 | 14.50 | 0.26 | 23.75 | 32.86 |
| J6 | Se | 280 | 0 | F | 12 | 7 | 121.90 | 4.40 | 18.90 | 0.23 | 27.02 | 34.69 |
| J11 | Se | 246 | 0 | M | 14 | 7 | 163.70 | 5.30 | 26.80 | 0.20 | 23.21 | 49.11 |
| J5 | Se | 301 | 1 |  |  |  |  |  |  |  |  |  |
| J1 | Se | 293 | 0 | M | 16 | 8 | 119.60 | 5.10 | 20.10 | 0.25 | 27.35 | 42.55 |
| J11 | Se | 248 | 1 |  |  |  |  |  |  |  |  |  |
| J1 | Se | 294 | 0 | M | 12 | 7 | 121.50 | 5.70 | 18.50 | 0.31 | 20.92 | 45.35 |
| J1 | Se | 295 | 0 | F | 12 | 8 | 107.20 | 4.30 | 19.90 | 0.22 | 26.12 | 38.82 |
| J11 | Se | 249 | 1 |  |  |  |  |  |  |  |  |  |
| J6 | Se | 281 | 0 | M | 12 | 8 | 105.70 | 4.10 | 17.80 | 0.23 | 22.92 | 46.67 |
| J9 | Se | 530 | 1 |  |  |  |  |  |  |  |  |  |
| J9 | Se | 540 | 0 | F | 16 | 6 | 116.40 | 4.70 | 16.50 | 0.28 | 27.39 | 61.29 |
| J5 | Se | 470 | 0 | M | 15 | 8 | 87.20 | 3.40 | 21.40 | 0.16 | 16.36 | 56.25 |
| J14 | Se | 420 | 0 | M | 15 | 6 | 109.70 | 4.60 |  |  | 25.19 |  |
| J14 | Se | 415 | 0 | M | 13 | 7 | 127.10 | 4.10 | 19.00 | 0.22 | 21.39 | 48.45 |
| J6 | Se | 550 | 1 |  |  |  |  |  |  |  |  |  |
| J11 | Se | 429 | 0 | M | 15 | 6 | 122.70 | 4.90 | 17.20 | 0.28 | 27.53 | 57.30 |
| J5 | Se | 440 | 1 |  |  |  |  |  |  |  |  |  |
| J7 | Se | 502 | 0 | M | 17 | 7 | 98.60 | 4.10 | 12.10 | 0.34 | 18.32 | 40.98 |
| J1 | Se | 425 | 0 | F | 16 | 6 | 102.50 | 4.70 | 13.90 | 0.34 | 20.70 | 36.11 |
| J5 | Se | 463 | 1 |  |  |  |  |  |  |  |  |  |
| J7 | Se | 486 | 1 |  |  |  |  |  |  |  |  |  |
| J7 | Se | 485 | 1 |  |  |  |  |  |  |  |  |  |
| J5 | Se | 454 | 1 |  |  |  |  |  |  |  |  |  |
| J6 | Se | 504 | 1 |  |  |  |  |  |  |  |  |  |
| J7 | Se | 498 | 0 | M | 15 | 8 | 117.00 | 3.90 | 17.90 | 0.22 | 19.99 | 49.50 |
| J11 | Se | 432 | 1 |  |  |  |  |  |  |  |  |  |
| J7 | Se | 501 | 1 |  |  |  |  |  |  |  |  |  |
| J6 | Se | 287 | 1 |  |  |  |  |  |  |  |  |  |
| J9 | Se | 539 | 0 | M | 17 | 7 | 120.80 | 4.10 | 19.70 | 0.21 | 22.37 | 52.33 |
| J7 | Se | 497 | 0 | F | 13 | 7 | 116.70 | 5.30 | 16.40 | 0.32 | 20.20 | 54.67 |
| J9 | Se | 524 | 1 |  |  |  |  |  |  |  |  |  |
| J9 | Se | 515 | 1 |  |  |  |  |  |  |  |  |  |
| J3 | Se | 554 | 1 |  |  |  |  |  |  |  |  |  |
| J7 | Se | 561 | 1 |  |  |  |  |  |  |  |  |  |
| J7 | Se | 568 | 1 |  | 16 |  | 113.80 |  |  |  |  |  |
| J7 | Se | 569 | 1 |  |  |  |  |  |  |  |  |  |
| J7 | Se | 563 | 1 |  |  |  |  |  |  |  |  |  |
| J7 | Se | 562 | 1 |  |  |  |  |  |  |  |  |  |
| J6 | Se | 584 | 0 | M | 17 | 7 | 106.40 | 4.50 | 15.80 | 0.28 | 16.97 | 72.41 |
| J14 | Se | 571 | 1 |  |  |  |  |  |  |  |  |  |
| J14 | Se | 418 | 1 |  |  |  |  |  |  |  |  |  |
| J3 | Se | 603 | 1 |  |  |  |  |  |  |  |  |  |
| J14 | Se | 579 | 0 | M | 12 | 7 | 131.70 | 5.90 | 19.20 | 0.31 | 21.55 | 48.31 |
| J14 | Se | 574 | 1 |  |  |  |  |  |  |  |  |  |
| J3 | Se | 595 | 1 |  |  |  |  |  |  |  |  |  |
| J22 | Se | 632 | 0 | F | 12 | 7 | 140.00 | 6.10 | 20.60 | 0.30 | 25.51 | 57.14 |
| J22 | Se | 683 | 1 |  |  |  |  |  |  |  |  |  |
| J7 | Se | 664 | 0 | M | 14 | 7 | 131.10 | 5.10 | 13.00 | 0.39 | 21.08 | 51.90 |
| J22 | Se | 635 | 1 |  |  |  |  |  |  |  |  |  |
| J19 | Se | 630 | 1 |  |  |  |  |  |  |  |  |  |
| J7 | Se | 665 | 1 |  |  |  |  |  |  |  |  |  |
| J22 | Se | 675 | 1 |  |  |  |  |  |  |  |  |  |
| J22 | Se | 689 | 0 | M | 14 | 6 | 133.60 | 3.80 |  |  | 20.54 |  |
| J22 | Se | 644 | 1 |  |  |  |  |  |  |  |  |  |
| J22 | Se | 659 | 1 |  |  |  |  |  |  |  |  |  |
| J22 | Se | 643 | 1 |  |  |  |  |  |  |  |  |  |
| J22 | Se | 696 | 1 |  |  |  |  |  |  |  |  |  |
| J19 | Se | 624 | 1 |  |  |  |  |  |  |  |  |  |
| J22 | Se | 702 | 1 |  |  |  |  |  |  |  |  |  |

**Table 2. Contact exposure experiment.**

| **M_ID** | **Treatment** | **Concentration (%)** | **L_ID** | **Mortality** |
| --- | --- | --- | --- | --- |
| A5 | T | 0 | 65 | 0 |
| A7 | T | 0 | 76 | 0 |
| A4 | T | 0 | 53 | 1 |
| A8 | T | 0 | 512 | 0 |
| A2 | T | 0 | 206 | 0 |
| A3 | T | 0 | 141 | 0 |
| A1 | T | 0 | 203 | 1 |
| A6 | T | 0 | 119 | 0 |
| A6 | T | 0 | 270 | 0 |
| A6 | T | 0 | 388 | 0 |
| A4 | T | 0.046 | 5 | 0 |
| A7 | T | 0.046 | 205 | 0 |
| A6 | T | 0.046 | 42 | 0 |
| A7 | T | 0.046 | 80 | 0 |
| A7 | T | 0.046 | 84 | 0 |
| A6 | T | 0.046 | 90 | 1 |
| A6 | T | 0.046 | 319 | 1 |
| A4 | T | 0.046 | 324 | 1 |
| A6 | T | 0.046 | 253 | 0 |
| A8 | T | 0.046 | 283 | 0 |
| A4 | T | 0.41 | 57 | 0 |
| A4 | T | 0.41 | 1 | 1 |
| A7 | T | 0.41 | 83 | 0 |
| A2 | T | 0.41 | 34 | 0 |
| A2 | T | 0.41 | 85 | 0 |
| A7 | T | 0.41 | 178 | 0 |
| A4 | T | 0.41 | 212 | 0 |
| A1 | T | 0.41 | 201 | 1 |
| A5 | T | 0.41 | 342 | 0 |
| A6 | T | 0.41 | 186 | 1 |
| A2 | T | 1.24 | 35 | 0 |
| A7 | T | 1.24 | 70 | 0 |
| A2 | T | 1.24 | 52 | 0 |
| A8 | T | 1.24 | 108 | 0 |
| A5 | T | 1.24 | 66 | 0 |
| A5 | T | 1.24 | 343 | 1 |
| A6 | T | 1.24 | 116 | 0 |
| A2 | T | 1.24 | 210 | 1 |
| A5 | T | 1.24 | 180 | 1 |
| A6 | T | 1.24 | 146 | 0 |
| A3 | T | 3.7 | 30 | 1 |
| A7 | T | 3.7 | 67 | 1 |
| A2 | T | 3.7 | 41 | 1 |
| A2 | T | 3.7 | 86 | 0 |
| A6 | T | 3.7 | 92 | 1 |
| A6 | T | 3.7 | 18 | 1 |
| A6 | T | 3.7 | 128 | 1 |
| A6 | T | 3.7 | 117 | 1 |
| A6 | T | 3.7 | 179 | 0 |
| A8 | T | 3.7 | 105 | 0 |
| A3 | T | 11.1 | 142 | 0 |
| A5 | T | 11.1 | 181 | 0 |
| A2 | T | 11.1 | 198 | 0 |
| A6 | T | 11.1 | 171 | 0 |
| A5 | T | 11.1 | 204 | 0 |
| A6 | T | 11.1 | 99 | 0 |
| A6 | T | 11.1 | 190 | 1 |
| A6 | T | 11.1 | 126 | 0 |
| A5 | T | 11.1 | 217 | 0 |
| A5 | T | 11.1 | 182 | 1 |
| A8 | T | 33.3 | 298 | 0 |
| A8 | T | 33.3 | 369 | 0 |
| A4 | T | 33.3 | 330 | 1 |
| A4 | T | 33.3 | 331 | 1 |
| A6 | T | 33.3 | 271 | 0 |
| A6 | T | 33.3 | 268 | 0 |
| A8 | T | 33.3 | 296 | 1 |
| A8 | T | 33.3 | 397 | 1 |
| A7 | T | 33.3 | 355 | 0 |
| A6 | T | 33.3 | 259 | 1 |
| A6 | T | 100 | 254 | 0 |
| A8 | T | 100 | 404 | 0 |
| A8 | T | 100 | 275 | 1 |
| A6 | T | 100 | 381 | 1 |
| A6 | T | 100 | 286 | 1 |
| A8 | T | 100 | 375 | 1 |
| A8 | T | 100 | 240 | 1 |
| A6 | T | 100 | 310 | 1 |
| A6 | T | 100 | 302 | 1 |
| A6 | T | 100 | 291 | 1 |
| A4 | S | 0 | 28 | 0 |
| A4 | S | 0 | 27 | 0 |
| A4 | S | 0 | 231 | 0 |
| A6 | S | 0 | 434 | 0 |
| A6 | S | 0 | 24 | 0 |
| A7 | S | 0 | 71 | 0 |
| A7 | S | 0 | 68 | 0 |
| A1 | S | 0 | 202 | 0 |
| A6 | S | 0 | 147 | 1 |
| A8 | S | 0 | 219 | 0 |
| A4 | S | 0.046 | 4 | 0 |
| A4 | S | 0.046 | 39 | 1 |
| A6 | S | 0.046 | 10 | 1 |
| A7 | S | 0.046 | 77 | 0 |
| A4 | S | 0.046 | 56 | 1 |
| A6 | S | 0.046 | 48 | 1 |
| A6 | S | 0.046 | 390 | 0 |
| A8 | S | 0.046 | 395 | 1 |
| A6 | S | 0.046 | 249 | 0 |
| A6 | S | 0.046 | 293 | 1 |
| A7 | S | 0.41 | 82 | 0 |
| A2 | S | 0.41 | 64 | 0 |
| A5 | S | 0.41 | 73 | 0 |
| A2 | S | 0.41 | 38 | 1 |
| A2 | S | 0.41 | 33 | 0 |
| A4 | S | 0.41 | 40 | 1 |
| A2 | S | 0.41 | 207 | 0 |
| A5 | S | 0.41 | 216 | 0 |
| A2 | S | 0.41 | 209 | 1 |
| A6 | S | 0.41 | 100 | 0 |
| A7 | S | 1.24 | 74 | 0 |
| A6 | S | 1.24 | 16 | 1 |
| A4 | S | 1.24 | 59 | 0 |
| A6 | S | 1.24 | 7 | 0 |
| A6 | S | 1.24 | 14 | 1 |
| A6 | S | 1.24 | 9 | 1 |
| A5 | S | 1.24 | 185 | 0 |
| A3 | S | 1.24 | 200 | 0 |
| A6 | S | 1.24 | 169 | 0 |
| A7 | S | 1.24 | 133 | 1 |
| A6 | S | 3.7 | 45 | 1 |
| A6 | S | 3.7 | 12 | 0 |
| A2 | S | 3.7 | 63 | 0 |
| A4 | S | 3.7 | 55 | 1 |
| A2 | S | 3.7 | 31 | 0 |
| A6 | S | 3.7 | 23 | 1 |
| A2 | S | 3.7 | 50 | 0 |
| A5 | S | 3.7 | 183 | 0 |
| A8 | S | 3.7 | 227 | 1 |
| A7 | S | 3.7 | 177 | 0 |
| A7 | S | 11.1 | 137 | 0 |
| A2 | S | 11.1 | 197 | 0 |
| A7 | S | 11.1 | 134 | 0 |
| A8 | S | 11.1 | 213 | 0 |
| A7 | S | 11.1 | 176 | 0 |
| A6 | S | 11.1 | 172 | 0 |
| A6 | S | 11.1 | 104 | 1 |
| A6 | S | 11.1 | 102 | 0 |
| A8 | S | 11.1 | 139 | 1 |
| A6 | S | 11.1 | 173 | 1 |
| A6 | S | 33.3 | 322 | 0 |
| A5 | S | 33.3 | 318 | 1 |
| A2 | S | 33.3 | 340 | 0 |
| A4 | S | 33.3 | 326 | 1 |
| A8 | S | 33.3 | 295 | 1 |
| A6 | S | 33.3 | 251 | 1 |
| A8 | S | 33.3 | 368 | 0 |
| A6 | S | 33.3 | 384 | 0 |
| A8 | S | 33.3 | 278 | 0 |
| A7 | S | 33.3 | 354 | 0 |
| A6 | S | 100 | 258 | 1 |
| A6 | S | 100 | 309 | 1 |
| A6 | S | 100 | 382 | 1 |
| A7 | S | 100 | 357 | 0 |
| A8 | S | 100 | 400 | 0 |
| A6 | S | 100 | 333a | 0 |
| A7 | S | 100 | 359 | 0 |
| A5 | S | 100 | 316 | 0 |
| A4 | S | 100 | 327 | 1 |
| A4 | S | 100 | 325 | 1 |
| A6 | Se | 0 | 49 | 0 |
| A2 | Se | 0 | 62 | 0 |
| A7 | Se | 0 | 69 | 0 |
| A4 | Se | 0 | 29 | 0 |
| A7 | Se | 0 | 341 | 0 |
| A4 | Se | 0 | 2 | 0 |
| A8 | Se | 0 | 282 | 1 |
| A8 | Se | 0 | 297 | 1 |
| A8 | Se | 0 | 280 | 0 |
| A6 | Se | 0 | 250 | 0 |
| A4 | Se | 0.046 | 54 | 1 |
| A5 | Se | 0.046 | 72 | 1 |
| A6 | Se | 0.046 | 22 | 1 |
| A4 | Se | 0.046 | 3 | 0 |
| A6 | Se | 0.046 | 311 | 0 |
| A6 | Se | 0.046 | 386 | 1 |
| A8 | Se | 0.046 | 276 | 0 |
| A6 | Se | 0.046 | 239 | 1 |
| A6 | Se | 0.046 | 320 | 1 |
| A6 | Se | 0.046 | 301 | 1 |
| A4 | Se | 0.41 | 58 | 0 |
| A6 | Se | 0.41 | 44 | 1 |
| A6 | Se | 0.41 | 8 | 0 |
| A2 | Se | 0.41 | 87 | 0 |
| A6 | Se | 0.41 | 93 | 1 |
| A6 | Se | 0.41 | 149 | 1 |
| A8 | Se | 0.41 | 346 | 1 |
| A8 | Se | 0.41 | 107 | 1 |
| A7 | Se | 0.41 | 175 | 0 |
| A6 | Se | 0.41 | 115 | 1 |
| A7 | Se | 1.24 | 75 | 1 |
| A6 | Se | 1.24 | 26 | 1 |
| A6 | Se | 1.24 | 20 | 0 |
| A2 | Se | 1.24 | 194 | 1 |
| A8 | Se | 1.24 | 345 | 1 |
| A8 | Se | 1.24 | 226 | 0 |
| A4 | Se | 1.24 | 154 | 0 |
| A2 | Se | 1.24 | 208 | 0 |
| A6 | Se | 1.24 | 123 | 0 |
| A5 | Se | 1.24 | 215 | 0 |
| A6 | Se | 3.7 | 11 | 1 |
| A6 | Se | 3.7 | 46 | 1 |
| A6 | Se | 3.7 | 21 | 1 |
| A2 | Se | 3.7 | 32 | 1 |
| A2 | Se | 3.7 | 196 | 0 |
| A6 | Se | 3.7 | 144 | 1 |
| A4 | Se | 3.7 | 155 | 1 |
| A8 | Se | 3.7 | 138 | 0 |
| A6 | Se | 3.7 | 122 | 1 |
| A4 | Se | 3.7 | 156 | 0 |
| A6 | Se | 11.1 | 127 | 1 |
| A6 | Se | 11.1 | 125 | 0 |
| A6 | Se | 11.1 | 121 | 0 |
| A8 | Se | 11.1 | 218 | 1 |
| A6 | Se | 11.1 | 101 | 1 |
| A6 | Se | 11.1 | 114 | 1 |
| A6 | Se | 11.1 | 111 | 1 |
| A6 | Se | 11.1 | 148 | 1 |
| A6 | Se | 11.1 | 143 | 1 |
| A6 | Se | 11.1 | 166 | 1 |
| A8 | Se | 33.3 | 242 | 1 |
| A6 | Se | 33.3 | 287 | 1 |
| A8 | Se | 33.3 | 274 | 0 |
| A6 | Se | 33.3 | 321 | 0 |
| A6 | Se | 33.3 | 323 | 1 |
| A6 | Se | 33.3 | 313 | 1 |
| A6 | Se | 33.3 | 387 | 0 |
| A5 | Se | 33.3 | 317 | 1 |
| A8 | Se | 33.3 | 399 | 1 |
| A6 | Se | 33.3 | 255 | 0 |
| A7 | Se | 100 | 360 | 0 |
| A6 | Se | 100 | 269 | 0 |
| A8 | Se | 100 | 364 | 1 |
| A7 | Se | 100 | 353 | 0 |
| A6 | Se | 100 | 307 | 1 |
| A6 | Se | 100 | 312 | 1 |
| A6 | Se | 100 | 261 | 0 |
| A6 | Se | 100 | 267 | 0 |
| A6 | Se | 100 | 333 | 1 |
| A6 | Se | 100 | 266 | 0 |

**References:**

[1] R Core Team. R: a language and environment for statistical computing. Vienna (Austria): R Foundation for Statistical Computing; 2023. Available from: https://www.R-project.org/
